# ZT-RIASE: Zero Trust-resilient identity attestation for securing smart industrial IoT environment

**DOI:** 10.1038/s41598-026-54343-0

**Published:** 2026-05-28

**Authors:** Rishita Verma, Gaurav Indra, Abhishek Kumar Pandey, Ashok Kumar Das, Vivekananda Bhat K

**Affiliations:** 1 Department of Information Technology, Indira Gandhi Delhi Technical University for Women, Kashmere Gate, 110006 Delhi, India; 2 Center for Security, Theory and Algorithmic Research, International Institute of Information Technology, 500032 Hyderabad, India; 3 Department of Computer Science and Engineering, College of Informatics, Korea University, 145 Anam-ro, Seongbuk-gu, 02841 Seoul, South Korea; 4 Manipal Institute of Technology, Manipal Academy of Higher Education, Manipal, Karnataka 576104 India

**Keywords:** Industrial Internet of Things (IIoT), Industry 5.0, Least privilege, Zero Trust, Identity attestation, Security, Engineering, Mathematics and computing

## Abstract

In the Industry 5.0 paradigm, collaborative intelligence, human–machine cooperation, and real-time cognitive automation have increased the dependence of industrial systems on secure and uninterrupted Industrial Internet of Things (IIoT) connectivity. However, this convergence also expands the cyberattack surface and exposes resource-constrained industrial devices to impersonation, replay, man-in-the-middle, rogue gateway, insider, and session-hijacking attacks. Existing authentication schemes mainly focus on initial access verification and often lack continuous Zero Trust enforcement, failure-resilient reconnection, and network-aware runtime validation. To address these limitations, this paper proposes ZT-RIASE, a Zero Trust-resilient identity attestation framework for securing smart industrial IoT environments. ZT-RIASE adopts a hybrid bootstrap–symmetric runtime design, where public-key cryptography is used only during initial device registration and key agreement, while recurring runtime identity attestation, session maintenance, reconnection, and continuous verification rely on lightweight symmetric-key and behavior-based mechanisms. The runtime protocol uses AES-128-GCM, hash/MAC-based integrity verification, nonce–timestamp freshness, and session-continuity tokens to ensure confidentiality, integrity, and replay resistance without repeated public-key operations. To further reduce recurrent authentication overhead, ZT-RIASE introduces Network-Aware Crypto-Behavioral Continuous Authentication (NA-CBCA), which verifies active sessions using token-use regularity, path/gateway consistency, command-access consistency, message-size deviation, request-rate behavior, packet-timing deviation, retransmission/error behavior, and energy/processing deviation. Timing-sensitive behavioral features are normalized using a network condition index based on RTT, jitter, packet loss, and retransmission rate, thereby reducing false positives under changing industrial network conditions. Performance evaluation using representative constrained-device profiles and ns-3 simulations demonstrates that runtime attestation requires 2.400 ms computation time, 0.625 KB communication overhead, 3.800 KB memory, and 1.998 mJ energy, while NA-CBCA requires only 0.350 ms, 64 bytes, 2.100 KB memory, and 0.246 mJ energy. Large-scale scalability analysis from 100 to 1000 IIoT devices further shows predictable aggregate overhead growth with stable per-device runtime delay. These results demonstrate that ZT-RIASE provides lightweight, failure-aware, and behavior-adaptive Zero Trust identity attestation suitable for realistic smart industrial IoT deployments.

## Introduction

The Industrial Internet of Things (IIoT) forms the foundation of smart manufacturing and industrial automation by enabling real-time sensing, industrial data communication, automated control, and continuous system monitoring^1,2^. With the growing deployment of IIoT technologies, notable gains have been achieved in system efficiency, condition monitoring, and coordination between cyber and physical components^3^. In addition, the close interaction among sensing units, actuation components, and industrial controllers has led to better use of available resources and higher productivity in manufacturing operations^2^. With the expansion of IIoT deployments in the industrial domains. The risk of security and privacy breaches has increased tremendously, thereby increasing the attack surface^4^. The high dependence on interconnected devices and services necessitates the need for strong defenses against data leakage and unauthorized access^5^. But, various state-of-the-art approaches provide partial protection and fail to handle recent adversarial and adaptive attacks^1^. Moreover, most existing authentication mechanisms mainly verify entities at the initial access stage and do not continuously validate the device identity throughout an active industrial session. This creates a security gap when a legitimate session is hijacked, a device changes its gateway path, or a compromised entity starts behaving abnormally after initial authentication. Also, Wireless IIoT (W-IIoT) systems further worsen this problem. W-IIoTs help with the mobility and the simplification of deployment^6^; their wireless nature exposes the industrial networks to interception, lateral intrusion, and sophisticated cyber attacks. Thereby, making the requirement for the improved security mechanisms more prominent^7,8^. In such dynamic wireless environments, authentication must also remain reliable under changing network conditions such as delay variation, jitter, packet loss, retransmissions, and temporary link disruption. Therefore, a practical IIoT security mechanism should not only authenticate devices, but should also support secure reconnection and runtime behavior-aware verification under unstable network conditions.

The integration of Artificial Intelligence (AI) into the Industrial Internet of Things (IIoT), also known as AI-IIoT, has helped in the advancement of the industrial automation process, enabling real-time control, improvement in the process adjustment, and predictive maintenance tasks^2^.. It improves the machine-to-machine (M2M) communication, reduces the dependence on human supervision, flexible industrial process, and also enables faster fault detection^3^. The AI-based data analysis also guides in identifying the platform failures and in resource optimization, thereby improving the system performance and increasing operational intelligence^2^. This frequent data exchange and the automated decisions increase the overall security risks in the AI-IIoT systems, including the malicious interference and data leakage^4^. The current security solutions cannot resolve the various attacks, such as data poisoning, rogue access points, or insider abuse, this leads AI-IIoT systems at risk of breach^1^.. Although symmetric-key methods are widely used to reduce computation cost, many protocols fail to provide the basic security features such as forward secrecy or adequate protection for temporary session keys^9,10^. These limitations necessitate the need for an identity attestation framework that is well-suited to meet the security requirements of AI-IIoT environments. In addition, recent behavioral-authentication schemes demonstrate the usefulness of continuous runtime verification, but most of them are designed for user-centric environments such as smartphones or non-portable computing devices. They generally do not integrate device-centric cryptographic attestation, network-aware behavior monitoring, and failure-resilient reconnection, which are required in smart industrial IIoT deployments.

### Research motivation

These security shortcomings motivate the need for the proposed ZT-RIASE framework, which strictly adheres to the Zero-Trust(ZT) principles given by the NIST for identity attestation by removing the assumed trust among the network entities and following the “never trust, always verify principle.” ZT-enforces continuous authentication and access control at each communication step^1^. In the ZT model, all the entities are presumed to be untrusted by default, irrespective of whether they are inside or outside the network premises. This lowers the unauthorized access, lateral movement, and privilege misuse in industrial systems^8^. ZT-RIASE uses the role-based access control (RBAC), context-aware authentication checks, and secure key confirmation to limit security risks related to key exposure and unauthorized entity actions^2,7^. Unlike the state-of-the-art, the proposed framework supports fast revocation of the access rights of any entity, which helps to stop compromised credentials or malicious behavior early^9^. By enforcing the ZT Network Policies (ZTNPs), ZT-RIASE applies least-privilege access, granting entities only the permissions required for their roles and thereby, reducing the overall attack surface^1,4^. ZT-RIASE follows a hybrid bootstrap–symmetric runtime design. Public-key cryptography is used only during initial device registration and key agreement, while recurring identity attestation, session maintenance, reconnection, and continuous verification are performed using lightweight symmetric-key and behavior-based mechanisms. This clarification separates the one-time registration cost from the recurring runtime cost and avoids repeated public-key operations during normal IIoT communication.

Furthermore, ZT-RIASE introduces Network-Aware Crypto-Behavioral Continuous Authentication (NA-CBCA), where the first authentication is performed using cryptographic identity attestation and subsequent verification within the same valid session is performed using behavior-aware runtime checks. NA-CBCA monitors token-use regularity, path/gateway consistency, command-access consistency, message-size deviation, request-rate behavior, packet-timing deviation, error/retransmission behavior, and energy/processing deviation. Timing-sensitive features are adjusted according to changing network conditions, while security-critical features such as token validity, gateway consistency, and command-access consistency remain strictly enforced.

### Research contributions

The proposed ZT-RIASE framework includes three primary entities: AI-enabled IIoT devices (AID), Edge-enabled Access Points (EAP), and Cloud-based Authentication Servers (CAS). These three entities form a layered security structure. AID operates as the smart industrial nodes with its adaptive security support, assisted by AI-based anomaly detection and intelligent cognitive abilities. EAPs serve as the nearby support edge nodes, which handle authentication and access checks of the AIDs that are closest to them. This helps in reducing the response delay. CAS operates as a centralized authentication server that verifies identities while protecting data integrity and confidentiality across the network. ZT-RIASE also provides a network failure-resistant fast re-connect feature without repeating the full verification process, which improves the session handling and further reduces the computation and signalling cost. The framework results are evaluated by performing simulations using Arduino Uno (ATmega328P) devices and network simulations in ns-3 (v3.41). The results indicate that ZT-RIASE reduces computation cost by 12.5% and communication overhead by 14.29% compared to the symmetric-key-based EAP-MAP protocol^9^. EAP-MAP is taken as the baseline due to its fair comparison with ZT-RIASE, as they both use symmetric key cryptography. The integration of ZT principles and symmetric key cryptography-based identity attestation ZT-RIASE provides an efficient and secure solution for AI-driven Industry 5.0 environments.

The main contributions of thus research work are as follows:

- *Network-Aware Crypto-Behavioral Continuous Authentication(NA-CBCA):* ZT-RIASE introduces a novel NA-CBCA mechanism, where the initial device interaction undergoes full cryptographic identity attestation, whereas subsequent interactions in an already validated session are verified through a lightweight behavior-consistency matrix. The NA-CBCA mechanism automatically adjusts the delay-sensitves attributes as per the ongoing network conditions, which mitigates the false positives that are cused by congestion, packet losss, and jitter in the network. Meanwhile, it also enforces the rigorous validation of tokens, routes, and command-access behavior.
- *Hybrid bootstrap with symmetric runtime attestation:* ZT-RIASE restricts the Public-Key Cryptographic(PKC) operations to the initial registration and key-agreement phase only. After registration is completed, the identity attestation, session security, and continuous trust affirmation are handled by using the lightweight symmetric key operations and behavior-aware validation. This approach minimizes the additional recurring cryptographic overhead while maintaining freshness guarantees, message integrity, and replay protection. The identity attestation phase uses AES-128-GCM with NIST-aligned nonce and timestamp lengths, resulting in lower computational cost compared with existing
- *ZT compliance:* ZT-RIASE abides by NIST ZT principles by performing repeated identity checks and providing time-limited access policies. It enforces RBAC and least-privilege policies that limit unauthorized actions and restrict lateral movement within the AI-IIoT environment.
- *Network Failure Resistant Reconnection:* ZT-RIASE provides support for quick reconnection process if there is any case of network failure or disruption in the environment. It enables the devices to reconnect without repeating the full authentication process if they reconnect within the allocated time frame. This reduces the delay time and improves the overall overhead, making it well-suited for the time-sensitive, resource-constrained Industry 5.0 environments.
- *Performance analysis:* ZT-RIASE achieves a lower computation cost by 12.5% and communication overhead by 14.29% compared with the symmetric-key EAP-MAP protocol^9^, while also reducing storage and energy overhead. These performance improvements are validated through comprehensive evaluation, including ns-3.41 simulations, real-time testbed experiments, and formal security analysis, demonstrating the suitability of ZT-RIASE for deployment in resource-constrained, AI-enabled IIoT environments.

## Literature review

Authentication schemes for IoT and IIoT systems proposed during the 2021–2026 period increasingly emphasize lightweight operation, privacy preservation, certificateless construction, and decentralized identity management to accommodate the constraints of heterogeneous cyber–physical infrastructures. However, existing schemes still fail to fully satisfy Zero Trust (ZT) requirements, including continuous verification, distrust-by-default operation, failure-resilient reconnection, and efficient identity attestation under real-time industrial conditions. Recent behavioral-biometric continuous authentication schemes further show that runtime behavior can be used to verify user or device legitimacy after initial authentication. However, most of these schemes are designed for smartphones, user terminals, or non-portable computing devices, and they do not jointly integrate cryptographic device attestation, industrial network awareness, and failure-resilient reconnection. Therefore, the related work is categorized into three primary groups: (i) lightweight mutual authentication protocols for IoT and IIoT, (ii) privacy-preserving, anonymous, and cross-domain identity-management schemes, and (iii) behavioral-biometric and continuous authentication schemes.

### Lightweight mutual authentication across IoT and IIoT domains

Many recent research works have proposed lightweight identity attestation frameworks and authentication protocols for domains such as industrial IoT, smart grids, healthcare systems, and fog-assisted IoT networks. For instance, Satpathy et al^11^. design a sustainable authentication mechanism for IoT–Fog–Cloud networks by optimizing computation, communication, and energy overheads. While effective for fog-assisted environments, the model maintains a traditional trust hierarchy and does not incorporate ZT features such as continuous verification or network-failure-aware re-attestation. Awaneesh et al^9^. improve EAP/AKA-based WLAN/5G authentication using client puzzles and stronger handshakes, but the scheme remains centralized and lacks multi-hop industrial support and disruption-aware re-authentication. Other lightweight schemes show similar gaps: drone-swarm authentication^12^ uses ASCON-AEAD and HMAC challenge-response but lacks ZT enforcement and hierarchical IIoT roles; ECC-based smart-grid authentication^13^ provides anonymity and mutual authentication but depends on costly point multiplication and a trusted authority; and edge-assisted VANET^14^ and robotics schemes^2^ reduce latency through offloading but still rely on trusted intermediaries and asymmetric cryptography.

WSN attestation schemes such as LMFA^15^ reduce cost through hash and bitwise operations, but mainly address user access rather than node-level trust or continuous identity validation. Similarly, distributed symmetric-key protocols^16^ and LCDMA^7^ lower overhead, yet provide limited support for dynamic identities, unstable links, continuous distrust modelling, and secure post-failure recovery. Hence, most lightweight authentication schemes remain access-centric and are less suitable for ZT-enabled smart industrial IIoT systems requiring repeated identity assurance and resilient re-authentication.

### Privacy-preserving, anonymous, and cross-domain authentication schemes

Another research direction addresses privacy, anonymity, and certificateless trust. Certificateless anonymous authentication^8^ removes certificate overhead and supports traceability, but its pairing-free public-key operations remain costly for constrained IIoT devices. Similarly, certificateless IIoT authentication^17^lowers certificate-management burden yet still requires asymmetric verification and lacks continuous authentication or ZT enforcement. Cross-domain anonymous schemes such as Luo et al^6^. provide unlinkability through ECC and pseudonyms, but target roaming IoT rather than hierarchical industrial networks. RFID authentication^18^ and ALMASH for healthcare IoT^19^ ensure anonymity and mutual authentication, but depend on backend/group-based workflows and do not support trust evolution, secure reconnection, or ZT re-attestation. Quantum-secure schemes such as QSKA^20^ introduce lattice/quantum-resilient primitives for privacy-preserving authentication, but their suitability for lightweight, continuous IIoT verification remains limited. While they demonstrate forward-looking cryptographic resilience, they do not model ZT threat assumptions, industrial role separations, or lightweight symmetric runtime attestation paths comparable to those required in IIoT.

It is observed that anonymous and privacy-oriented authentication approaches are effective in protecting against linkability and removing the need for conventional digital certificates. However, they do not combine these properties with repeated identity validation, runtime behavioral verification, and ZT principles. They also do not provide network-failure-resistant authentication in cases of link interruption or session disruption. Additionally, many privacy-preserving schemes rely heavily on public-key cryptographic operations, which increases processing delay and makes them less suitable for industrial environments with strict latency requirements.

### Behavioral-biometric and continuous authentication schemes

Recent behavioral-biometric authentication schemes aim to overcome the limitation of one-time authentication by continuously monitoring user or device behavior during an active session. Ayeswarya et. al^21^. provides a broad review of continuous authentication methods based on physiological biometrics, behavioral biometrics, multimodal biometrics, and context-aware authentication.It shows that continuous authentication improves post-login security, but practical issues remain, and it does not provide an IIoT-specific cryptographic attestation or failure-aware reconnection framework.

Raja et. al^22^. proposes a behavioral-biometrics-based certificateless authentication and key-agreement protocol for advanced metering infrastructure. It uses static and dynamic behavioral features such as received signal strength, recurrent communication rate, residual power, distance from access point, and MAC address to bind identity information and mitigate man-in-the-middle attacks. This work is relevant because it demonstrates that device behavior can strengthen authentication in smart-grid environments. However, it still relies on ECC-based authentication and key agreement, and it does not provide a ZT runtime mechanism for continuous behavior adaptation, network-aware behavioral scoring, or failure-resilient reconnection. Shen et. al^23^. proposes a semantic-aware multimotion behavioral biometric authentication system for smartphones. It uses adaptive segmentation of multimotion sensor data, causal temporal convolutional representation, and a multicenter deep one-class classifier to improve implicit authentication under real-world smartphone usage. This work is relevant because it supports behavior-aware segmentation and one-class modeling for continuous authentication, but it remains smartphone-centric and lacks cryptographic device attestation, IIoT identity management, ZT enforcement, and failure recovery. Lee et al^24^. applies keystroke dynamics and TPM support for ZT-based continuous authentication, but it targets user behavior in general computing environments rather than constrained IIoT identity attestation, network-aware verification, or reconnection under unstable links. Shen et. al^25^. handles behavior drift using touch, motion, and incremental learning, yet it does not include cryptographic bootstrap, symmetric runtime attestation, ZT policy control, or industrial failure handling. Li et. al^26^. explores multimodal behavioral fusion, but it is not designed for IIoT-oriented cryptographic attestation or resilient Zero Trust authentication. It combines contextual behavior, mouse behavior, and information-interaction behavior using data augmentation, LSTM-autoencoder-based anomaly detection, and stacked decision-level fusion. This work demonstrates that combining multiple behavioral modalities can improve authentication robustness compared with unimodal behavior authentication. However, it is designed for non-portable human-computer interaction systems and does not address industrial IoT device attestation, cryptographic session maintenance, or failure-resilient reconnection.

Therefore, behavioral-biometric authentication schemes demonstrate the feasibility of continuous runtime verification, but they are not sufficient alone for smart industrial IIoT security. Most behavior-based schemes focus on user behavior on smartphones, desktops, or web-based systems, whereas IIoT requires device-centric identity attestation, lightweight runtime cryptography, gateway-assisted enforcement, unstable-link handling, and policy-bound reconnection. This motivates the proposed Network-Aware Crypto-Behavioral Continuous Authentication (NA-CBCA), in which ZT-RIASE performs cryptographic identity attestation during initial registration or session establishment and then uses network-aware behavioral verification for repeated runtime authentication within the same valid session.

### Recent advances in post-quantum authentication and secure IIoT systems

Recent research has increasingly focused on strengthening authentication mechanisms for emerging IoT and industrial cyber–physical environments through the integration of advanced cryptographic techniques, decentralized trust infrastructures, and privacy-preserving identity mechanisms. Several recent studies have explored authentication frameworks that combine blockchain-based identity verification, hardware-rooted trust primitives such as physically unclonable functions (PUFs), and lightweight cryptographic mechanisms to enhance device authenticity and network resilience^27^. Recent works have explored post-quantum primitives such as ML-KEM and ML-DSA in TLS-based IoT authentication^28^, ECC-based multi-phase authentication for cloud-assisted IoT^29^, and blockchain-enabled identity management with MFA, RBAC/ABAC, and post-quantum signatures^30^. Other studies use lattice-based cryptography, pseudonymization, and federated learning for privacy-preserving authentication^31^, while group-based authentication reduces signaling and bandwidth overhead in dynamic networks^32^.

Fatima et al^33^. study IoT-enabled intelligent production systems involving sensors, autonomous devices, and cloud services for monitoring, predictive maintenance, and warehouse automation. Their work shows the operational value of connected industrial IoT, but also highlights security concerns related to authentication, identity management, and unauthorized access.

Although these studies improve authentication, privacy, decentralization, and post-quantum readiness, they mostly address isolated security aspects. In contrast, ZT-RIASE targets resilient Zero-Trust identity attestation for smart industrial IoT by combining continuous verification, adaptive trust enforcement, lightweight runtime attestation, network-aware behavioral authentication, and failure-resilient reconnection on constrained devices.

### Identified research gap

The above review shows that existing authentication protocols can be broadly divided into three categories. The first category includes lightweight authentication protocols that reduce computation and communication overhead, but generally do not comply with ZT principles or continuous verification requirements. The second category includes privacy-preserving, anonymous, certificateless, blockchain-assisted, and post-quantum authentication schemes that improve identity privacy and cryptographic robustness, but often rely on heavier public-key operations and do not address network-failure-resilient session recovery. The third category includes behavioral-biometric continuous authentication schemes that verify user behavior during runtime, but they are mostly designed for smartphones, desktops, or user-centric environments rather than smart industrial IIoT device attestation.

Therefore, a clear research gap exists in designing an authentication framework that jointly supports cryptographic identity attestation, lightweight runtime verification, behavior-aware continuous authentication, network-aware decision-making, failure-resilient reconnection, and ZT policy enforcement. No prior work sufficiently integrates public-key-based initial registration, symmetric-key runtime attestation, behavioral continuous authentication, and secure recovery after unstable industrial network conditions. The absence of such an integrated design motivates the proposed ZT-RIASE framework, which introduces hybrid bootstrap with lightweight symmetric runtime attestation and Network-Aware Crypto-Behavioral Continuous Authentication (NA-CBCA). The research gaps are summarized in Table 1.

**Table 1:** Comparative positioning of ZT-RIASE with recent authentication and behavioral-biometric continuous authentication schemes.

Scheme/Study	Main technique	CV	BBA	NAD	NFR	ZT	Main limitation
Satpathy et al^11^.	Lightweight IoT–Fog–Cloud authentication	No	No	No	No	Partial	Optimizes computation and energy cost, but lacks continuous verification and failure-aware re-authentication.
Salem et al^13^.	ECC-based smart-grid authentication	No	No	No	No	Partial	Provides anonymity and mutual authentication, but incurs public-key computation overhead.
Illyass et al^12^.	ASCON and HMAC-based authentication	No	No	No	No	No	Lightweight for drone-swarm networks, but lacks ZT-oriented industrial access enforcement.
QSKA^20^	Quantum-secure key agreement	No	No	No	No	Partial	Provides cryptographic resilience, but does not address runtime behavioral verification or reconnection.
RFID–ECC^18^	ECC-based RFID authentication	No	No	No	No	No	Focuses on RFID anonymity and authentication, but lacks continuous identity attestation.
LMFA–WSN^15^	Hash and bitwise authentication	No	No	No	No	No	Efficient for constrained devices, but limited to access authentication without ZT-based continuous checks.
EAP-based IoT^9^	EAP/AKA-based authentication	No	No	No	Partial	Partial	Strengthens initial authentication but does not support behavior-aware continuous authentication.
ALMASH^19^	ECC and Shamir secret sharing	No	No	No	No	Partial	Provides anonymity and group authentication, but lacks ZT-based re-attestation and reconnection support.
LCDMA^7^	Symmetric algebraic authentication	No	No	No	No	Partial	Reduces cryptographic cost, but lacks continuous verification and failure-resilient session recovery.
Certificateless IIoT^17^	Certificateless public-key authentication	No	No	No	No	Partial	Reduces certificate-management overhead, but still depends on asymmetric verification.
Certificateless anonymous scheme^8^	Anonymous mutual authentication	No	No	No	No	Partial	Supports privacy and traceability, but is relatively costly for constrained IIoT runtime use.
Luo et al^6^.	ECC and pseudonym-based cross-domain authentication	No	No	No	No	Partial	Supports roaming anonymity, but is not designed for hierarchical industrial ZT deployment.
Blockchain-MFA-ABAC scheme^30^	Blockchain, MFA, ABAC, and PQC	Partial	No	No	No	Yes	Strong access control, but relatively heavy for constrained runtime IIoT authentication.
PQC/TLS-based IoT authentication^28^	Post-quantum TLS authentication	No	No	No	No	Partial	Provides quantum resistance, but may increase computation and message-size overhead.
Group authentication scheme^32^	Group authentication and key agreement	No	No	No	Partial	Partial	Reduces signaling overhead, but lacks individual behavior-aware continuous verification.
Privacy-preserving FL/pseudonym scheme^31^	Lattice cryptography, pseudonyms, and FL	Partial	Partial	No	No	Partial	Enhances privacy and scalability, but does not support failure-aware session continuity.
Blockchain/PUF-based authentication^27^	Blockchain and PUF-based authentication	Partial	No	No	No	Partial	Improves device authenticity, but increases storage, communication, and deployment overhead.
Ayeswarya et. al^21^.	Biometric-based continuous authentication survey	Yes	Yes	Partial	No	Partial	Reviews physiological, behavioral, multimodal, and context-aware CA, but does not propose an IIoT-specific cryptographic attestation framework.
Raja et al^22^.	Behavioral biometrics with ECC-based AKA	Partial	Yes	Partial	No	Partial	Mitigates MITM using behavioral identity binding, but still depends on ECC and lacks ZT runtime behavior adaptation.
Shen et al^23^.	Semantic-aware multimotion implicit authentication	Yes	Yes	Partial	No	Partial	Provides strong multimotion behavioral authentication, but is smartphone-focused and lacks cryptographic IIoT attestation.
Lee et al^24^.	Keystroke-based ZT continuous authentication	Yes	Yes	Partial	No	Yes	Supports ZT-based user CA, but focuses on user keystroke behavior rather than industrial device attestation and failure recovery.
Shen et al^25^.	Touch behavior with incremental learning	Yes	Yes	Partial	No	Partial	Handles behavior drift over time, but lacks cryptographic bootstrap, ZT enforcement, and IIoT reconnection support.
Li et al^26^.	Contextual, mouse, and information-interaction behavior fusion	Yes	Yes	Partial	No	Partial	Improves CA through multimodal fusion, but does not address IIoT identity attestation or failure-resilient reconnection.
**ZT-RIASE with NA-CBCA**	**PKC bootstrap, AES-128-GCM runtime attestation, NFR, and network-aware behavior matrix**	**Yes**	**Yes**	**Yes**	**Yes**	**Yes**	**Integrates cryptographic attestation, behavior-aware continuous authentication, network-aware decision-making, and failure-resilient reconnection for smart industrial IoT.**

**Abbreviations:** CV: Continuous Verification; BBA: Behavior-Based Authentication; NAD: Network-Aware Decision; NFR: Network-Failure-Resilient Reconnection; ZT: Zero Trust; CA: Continuous Authentication; AKA: Authentication and Key Agreement; PKC: Public-Key Cryptography; FL: Federated Learning.

## System model

This section provides the details of the Network Model, Adversary Model, and Security Objectives.

### Network model

**Fig. 1:**
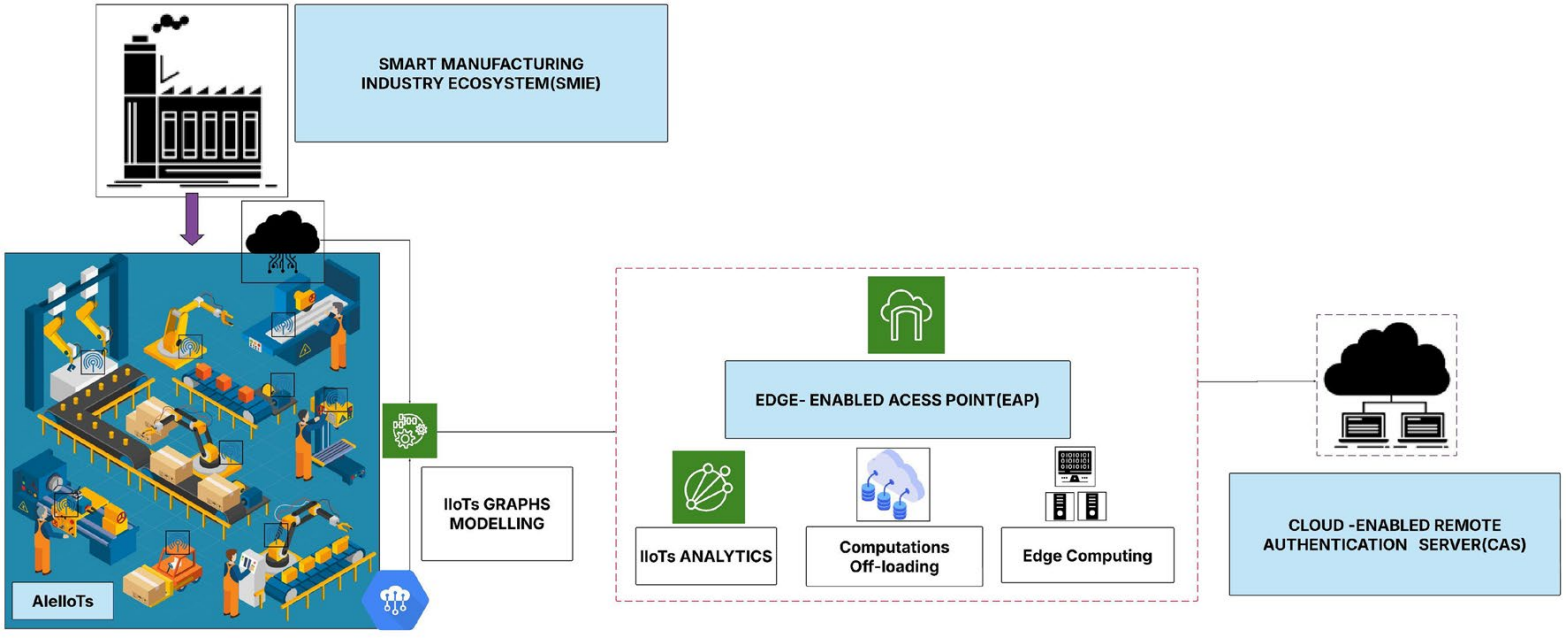
Network Model for AI-enabled IIoT.

The network setup with all the entities is illustrated in Fig. 1, enabling industrial devices to securely access the network services in the Smart Manufacturing Industrial setting. The main entities of the network are mentioned below in detail:

- *AI-enabled IIoT Devices (AID):* The devices are intelligent, which are equipped with sensors, actuators, and local processing capabilities. By integrating embedded AI and adaptive communication features, they enable autonomous operation support, efficient resource use, and automated control in smart factories.
- *Edge-enabled Access Points (EAP):* EAPs operate at the network edge and act as gateways between the AID and CAS. They perform local attestation, data processing, and have decision support, which helps reduce delay and supports near real-time operation.
- *Cloud-enabled Authentication Server (CAS):* CAS is a centrally managed server that verifies device identities and controls access permissions. It is hosted on the cloud and supports authentication for on-site industrial networks.

**Fig. 2:**
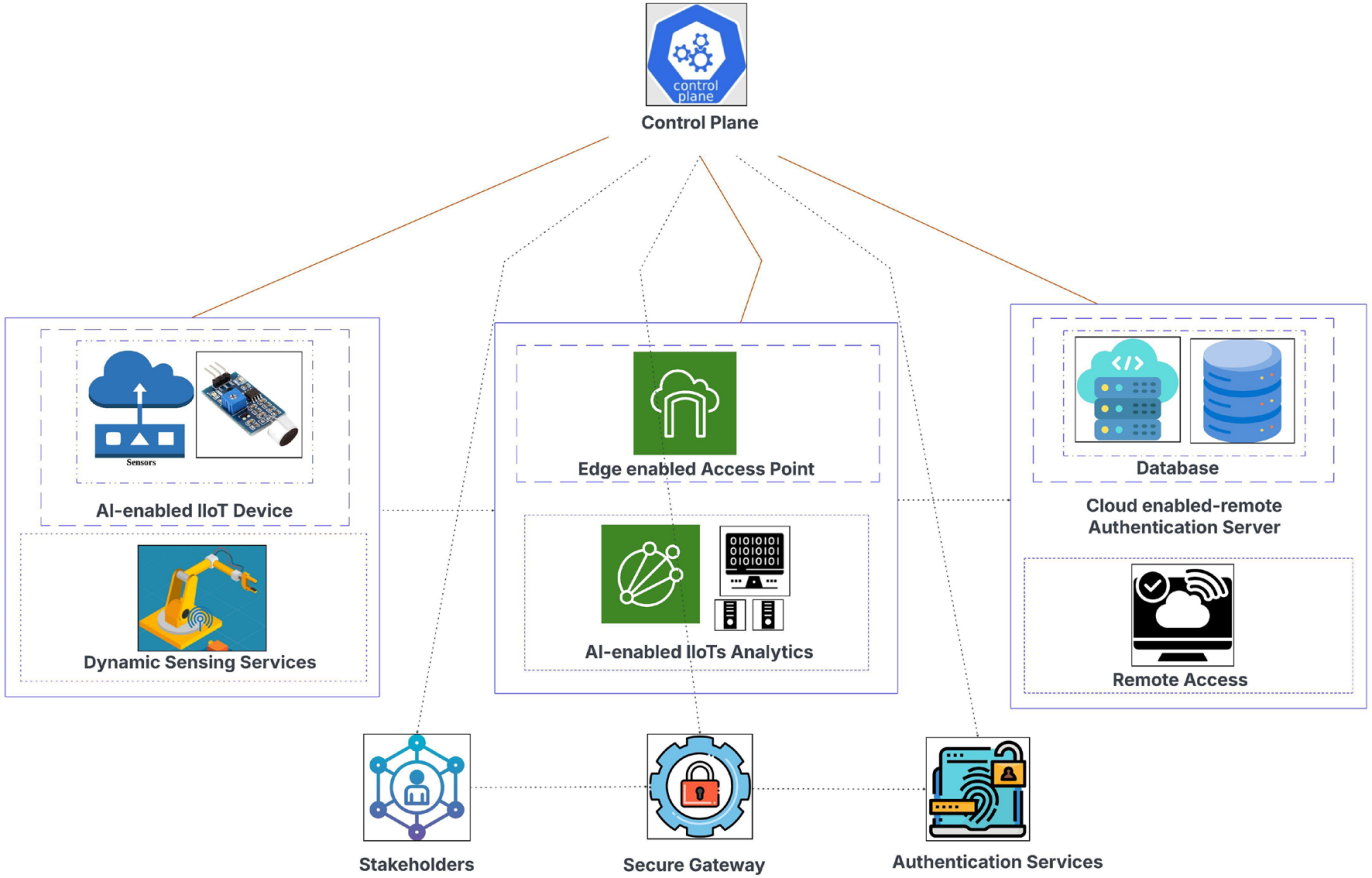
Zero Trust Architecture.

Figure 2 illustrates the NIST compliant-Zero Trust Architecture (ZTA) adopted in the proposed ZT-RIASE, to maintain secure and continuous authentication among AID, EAP, and CAS. Unlike the conventional perimeter-based models, the framework verifies the identities at each point of communication, which limits unauthorized access and identity misuse. The authentication requests pass through EAPs and are confirmed by the CAS using device credentials, past activity, and defined security rules. By ensuring adaptive security and real-time decision-making, ZT-RIASE provides a resilient framework.

#### Protocol design clarification

ZT-RIASE is a fully symmetric-key-based identity attestation framework. Earlier references to ECDH or elliptic-curve-based ephemeral secret exchange were artifacts of comparing ZT-RIASE with prior asymmetric schemes and are not part of the proposed protocol. All online operations in ZT-RIASE rely exclusively on symmetric primitives, namely AES-128, hash-based freshness validation, timestamps, and nonces. No Diffie–Hellman or public-key operation appears in the attestation or reconnection phases.

#### Roles and responsibilities

To ensure internal consistency, the roles in ZT-RIASE are defined as follows:

- **AI-enabled IIoT Device (AID):** It generates fresh nonce, timestamps, and initiates attestation. Performs lightweight symmetric encryption and MAC verification.
- **Edge Access Point (EAP):** It acts only as a forwarding and rate-limiting intermediary. EAP does not participate in cryptographic verification or key generation.
- **Cloud Authentication Server (CAS):** It verifies attestation transcripts, validates freshness, generates session identifiers, and issues the final session key.

All protocol diagrams, tables, and message exchanges have been aligned to reflect this role separation.

### Adversary model

The proposed framework is evaluated by using the Dolev–Yao (DY) adversary model^4^ and the Canetti–Krawczyk (CK) threat model^34^. Under these referred models, the adversary is assumed to have the following capabilities.

- The adversary has complete control over open wireless channels, including the ability to intercept, modify, block, or inject messages.
- The adversary has restricted computational capability, such that only one secret value can be guessed within feasible time limits.
- The adversary can capture and analyze messages from multiple protocol runs, to attempt linking or tracking.
- The adversary can act as an intermediary between communicating entities and perform man-in-the-middle attacks.

### Security objectives

The proposed identity attestation framework, ZT-RIASE, is designed to meet the following security goals:

- *Mutual Anonymous Identity Validation:* ZT-RIASE supports anonymous identity attestation among AID, EAP, and the CAS, while defending against impersonation, replay attacks, man-in-the-middle attacks, and insider abuse.
- *Network Access Protection:* The framework prevents unauthorized access to the network and mitigates threats such as denial-of-service attacks, device tracing, spoofing, scanning, and intrusion attempts.
- *Cryptographic Protection:* The scheme applies cryptographic safeguards to protect against interception, message alteration, unauthorized data use, and to maintain forward and backward secrecy.
- *Cyber Defense Capability:* The framework protects against malware threats, unauthorized disclosure of data, and cyber attacks targeting industrial communication systems.

## The proposed Zero-Trust anonymous identity attestation framework

This section overviews this research’s foundational concepts and background information. In this section, $A \rightarrow B$: *msg* denotes the message *msg* is sent by the entity *A* to the entity *B*, $E_k: P \rightarrow C$ denotes the encryption of plaintext using the key *k* to get the ciphertext *c* by symmetric key encryption, $D_k: C \rightarrow P$ denotes decryption of ciphertext using the key *k* to get plaintext *p* by symmetric key encryption, and *H*(*m*) denotes hash of the message *m*, where $H(\,)$ is a cryptographic one-way collision-resistant hash function. Table 2 provides the various notations and their meanings.

### Canonical protocol messages

For coherence across the manuscript, all figures, tables, BAN logic (Burrows-Abadi-Needham logic), Scyther models, and performance evaluations follow the canonical attestation flow.

**Phase 1: Initial Device Registration (offline)** It is performed once, and it is not part of runtime authentication.

**Phase 2: Mutual Attestation and Session-Key Establishment** This phase involves the exchange of the following messages:

- **M1:** $$AID \rightarrow CAS: \{T_1, cnonce_2\}_{k}$$
- **M2:** $$CAS \rightarrow AID: \{T_2, nonce_4\}_{k}$$
- **M3:** $$AID \rightarrow CAS: \{T_3, nonce_4\}_{k}$$
- **M4:** $$CAS \rightarrow AID: \{T_4, SK\text {-binding fields}\}_{k}$$

**Phase 3: Network-Failure-Resilient (NFR) Reconnection**

In this phase, the following message exchanges take place:

- **R1:** $$AID \rightarrow CAS: \{T_5, cnonce'_2\}_{k}$$
- **R2:** $$CAS \rightarrow AID: \{T_6, nonce'_4, sess\_id^{new}\}_{k}$$

**Table 2:** List of Abbreviations.

Notation	Definition
$n_{\text {CAS}}$	Nonce generated by CAS using a Pseudo Random Number Generator (PRNG).
$T^{\text {EAP}}$	Timestamp obtained by EAP using its synchronized local clock.
$cn^{\text {EAP}}$	Nonce generated by EAP using PRNG.
*C*	Ciphertext computed by EAP using public key cryptography (PKC).
*PW*	Password of any entity used for authentication.
$K_{\text {CAS}}$	Public key of CAS used in encryption.
*M*	Message containing encrypted credentials sent from EAP to CAS.
$T^{\text {CAS}}$	Timestamp obtained by CAS to verify the freshness of the authentication request.
$\Delta T$	Maximum allowable threshold time difference for freshness verification.
*AP*	Unique identifier of EAP used in authentication.
$X_{\text {UID}}$	Pseudo-anonymous identity of EAP stored by CAS.
$sess\_id$	Unique session identifier assigned by CAS.
$Z_{\text {UID}}$	Pseudo-anonymous identity of AID stored by CAS.
*k*	Short-lived key valid only for a session.
$n_i$	Pseudorandom nonce used in different authentication steps.
*SK*	Long-term session key computed for secure communication.
*LT*	Lease time defining the duration of the session key validity.
*TSK*	Temporary session key computed for fast reconnection.
$AS_{\text {ID}}$	Unique identifier of CAS.
$ID_{\text {new}}$	New temporary identifier assigned for fast reconnection.
*CH*	Ciphertext used in authentication messages.
*RCH*	Response ciphertext sent by CAS for verification.
*RES*	Response message from AID containing authentication confirmation.
*CHF*	Final encrypted message containing the session key.

### Registration phase

This phase describes the registration setup between (i) Edge-enabled Access point (EAP) and Cloud-enabled remote Authentication server (CAS), (ii) AI-enabled IIoT Device (AID) and Cloud-enabled remote Authentication server (CAS).

**Fig. 3:**
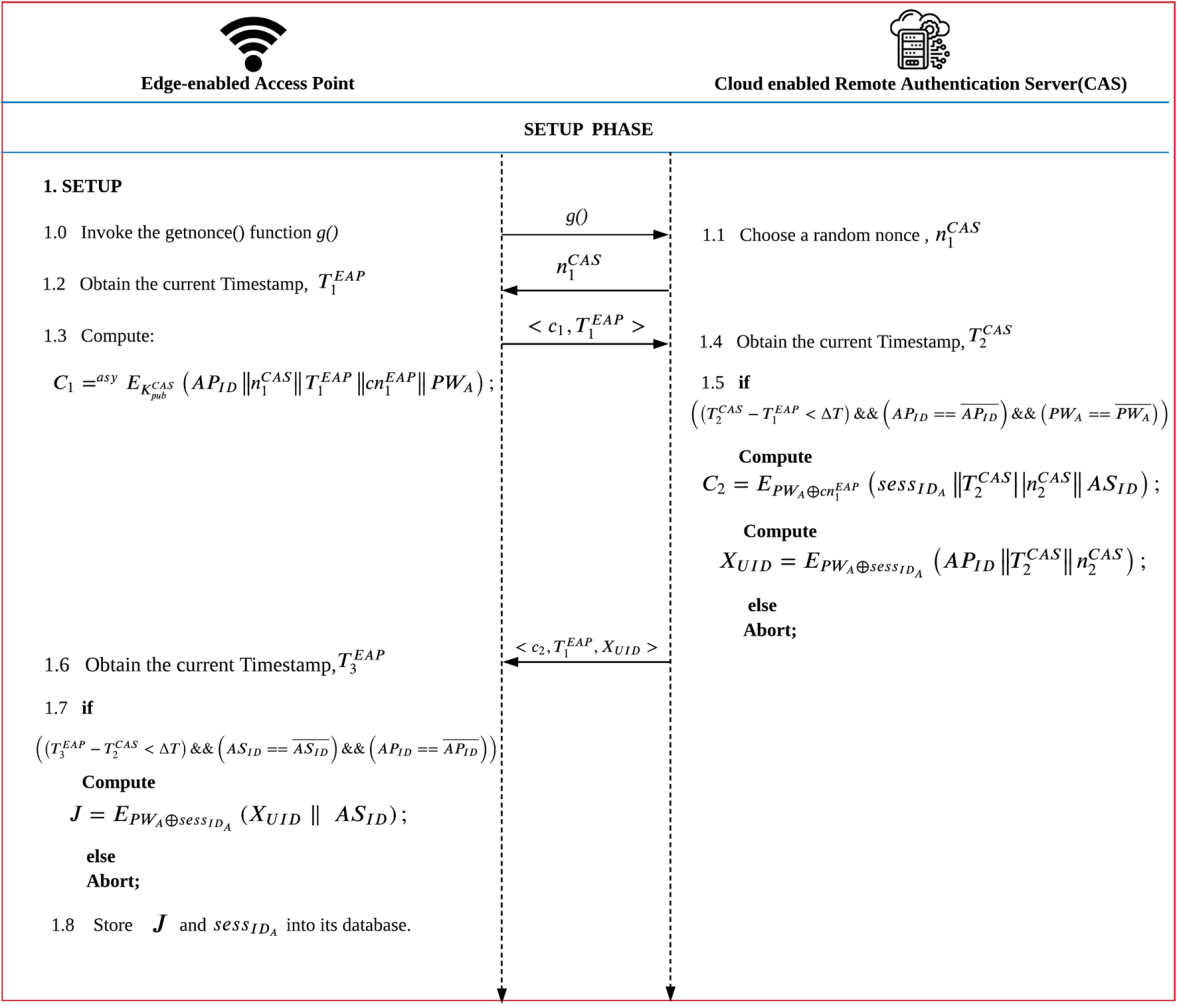
Registration between EAP and CAS.

**Fig. 4:**
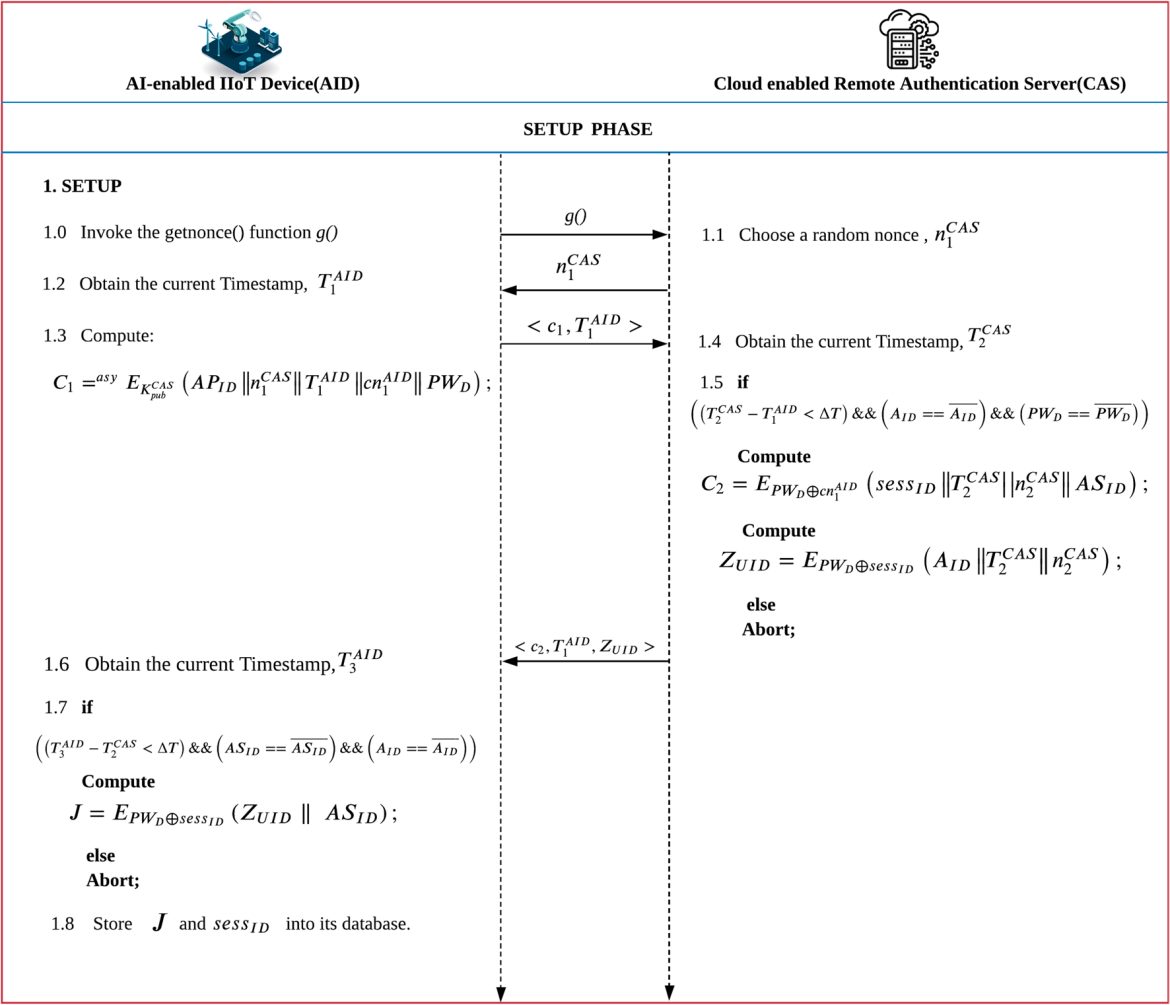
Registration between AID and CAS.

- **Registration between EAP and CAS:** It involves the following steps:
  - $EAP \rightarrow CAS$: EAP initiates the registration process and requests CAS to begin the registration setup shown in Fig. 3.
  - $CAS \rightarrow EAP$: CAS receives the registration request from EAP and generates a pseudorandom nonce $n_1^{CAS}$ using a Pseudo Random Number Generator (PRNG). CAS sends this nonce $n_1^{CAS}$ to EAP.
  - $EAP \rightarrow CAS$: After receiving the nonce $n_1^{CAS}$, EAP obtains the current timestamp $T_1^{EAP}$ using its synchronized local clock and generates a pseudorandom nonce $cn_1^{EAP}$ using a PRNG. EAP computes the ciphertext $C_1 = E_{K^{CAS}}^{pub}(AP_{ID} \parallel n_1^{CAS} \parallel T_1^{EAP} \parallel cn_1^{EAP} \parallel PW_A)$ using its unique identifier $AP_{ID}$, the nonce $n_1^{CAS}$, timestamp $T_1^{EAP}$, generated nonce $cn_1^{EAP}$, and password $PW_A$ encrypted using CAS’s public key $K^{CAS}_{pub}$ with public key cryptography (PKC). EAP then sends the message $M_1 = \langle C_1, T_1^{EAP} \rangle$ to CAS.
  - $CAS \rightarrow EAP$: Upon receiving message $M_1$, CAS obtains the current timestamp $T_2^{CAS}$ and checks the freshness condition $T_r - T_s < \Delta T$, where $T_r$ is the receiver’s timestamp, $T_s$ is the sender’s timestamp, and $\Delta T$ is the allowable threshold. This becomes $T_2^{CAS} - T_1^{EAP} < \Delta T$. If true, CAS decrypts $C_1$ using its private key $K^{CAS}_{pri}$. Upon decryption, CAS retrieves the parameters $\langle AP_{ID}, n_1^{CAS}, T_1^{EAP}, cn_1^{EAP}, PW_A \rangle$ and verifies them against its credential database. If the condition $(T_1^{EAP} == T_1^{EAP'}) \wedge (AP_{ID} == AP_{ID'}) \wedge (PW_A == PW_A')$ holds, then CAS registers the EAP.
  - CAS securely stores EAP’s encrypted identity as $X_{UID} = E_{sess\_id_A \oplus cn_1^{EAP}}(AP_{ID} \parallel T_2^{CAS} \parallel n_2^{CAS})$, where $sess\_id_A$ is the session ID assigned by CAS and $n_2^{CAS}$ is a newly generated nonce.The XOR-based derivation preserves secrecy, limits key repetition, increases data privacy, and counters replay attacks based on exploitation.
  - CAS then computes $C_2 = E_{PW_A \oplus cn_1^{EAP}}(AS_{ID} \parallel sess\_id_A \parallel T_2^{CAS} \parallel n_2^{CAS})$ and sends $M_2 = \langle C_2, T_2^{CAS}, X_{UID} \rangle$ to EAP.
  - *EAP*: Upon receiving $M_2$, EAP obtains timestamp $T_3^{EAP}$ and checks freshness. If the condition is valid, EAP decrypts $C_2$ to retrieve parameters $\langle AS_{ID}, sess\_id_A, T_2^{CAS}, n_2^{CAS} \rangle$. It checks the correctness confirms correctness by verifying $(T_2^{CAS} == T_2^{CAS'}) \wedge (AS_{ID} == AS_{ID'})$. If true, EAP further decrypts $X_{UID}$ to obtain $\langle AP_{ID}, T_2^{CAS}, n_2^{CAS} \rangle$, and verifies $(T_2^{CAS} == T_2^{CAS'}) \wedge (AP_{ID} == AP_{ID'})$.
  - After successful validation, EAP registers the identity of CAS and computes $L = E_{sess\_id_A \oplus PW_A}(X_{UID}, AS_{ID})$. It stores *L* in its database for use in the upcoming authentication phase.
- **Registration between EAP and CAS:** The steps of this phase are listed below:
  - $AID \rightarrow CAS$: The AID starts the registration process by sending a request to the CAS to set up registration, as shown in Fig. 4.
  - $CAS \rightarrow AID$: CAS receives the registration request from AID and generates a pseudorandom nonce $n_1^{CAS}$ via a PRNG. CAS sends this nonce $n_1^{CAS}$ to AID.
  - $AID \rightarrow CAS$: After receiving the nonce $n_1^{CAS}$, AID obtains the current timestamp $T_1^{AID}$ using its synchronized local clock and generates a pseudorandom nonce $cn_1^{AID}$ via a PRNG. AID computes the ciphertext $G_1 = E_{K^{CAS}_{pub}}^{PKC}(A_{ID} \parallel n_1^{CAS} \parallel T_1^{AID} \parallel cn_1^{AID} \parallel PW_D)$ using its unique identifier $A_{ID}$, the received nonce $n_1^{CAS}$, timestamp $T_1^{AID}$, its generated nonce $cn_1^{AID}$, and password $PW_D$, encrypted using CAS’s public key $K^{CAS}_{pub}$ via PKC. AID also computes the hash $H = H(G_1 \oplus n_1^{CAS} \oplus cn_1^{AID} \oplus PW_D)$. AID sends the message $M_1 = \langle G_1, T_1^{AID}, H \rangle$ to CAS.
  - $CAS \rightarrow AID$: After receiving $M_1$, CAS obtains the current timestamp $T_2^{CAS}$ and verifies the freshness condition. If the condition $T_2^{CAS} - T_1^{AID} < \Delta T$ holds, CAS decrypts $G_1$ using its private key $K^{CAS}_{pri}$ via PKC; otherwise, the message is discarded. After decryption, CAS retrieves the parameters $\langle A_{ID}, n_1^{CAS}, T_1^{AID}, cn_1^{AID}, PW_D \rangle$. CAS verifies these parameters against its credential database. If the condition $(T_1^{AID} == T_1^{AID'}) \wedge (A_{ID} == A_{ID'}) \wedge (PW_D == PW_D') \wedge (H == H')$ is true, then CAS registers the AID.
  - After successful registration, CAS stores AID’s credentials in encrypted form as $Z_{UID} = E_{sess\_id \oplus cn_1^{AID}}(A_{ID} \parallel T_2^{CAS} \parallel n_2^{CAS})$, where $sess\_id$ is a session identifier assigned by CAS, $n_2^{CAS}$ is a newly generated nonce, and the key is derived using XOR between $sess\_id$ and $cn_1^{AID}$. This process protects data secrecy, avoids repeated key use, preserves integrity and authenticity, and defends against replay attacks.
  - CAS then computes the ciphertext $G_2 = E_{PW_D \oplus cn_1^{AID}}(AS_{ID} \parallel sess\_id \parallel T_2^{CAS} \parallel n_2^{CAS} \parallel k)$, where $AS_{ID}$ is the unique identifier of CAS, and *k* is a short-lived session key. CAS sends the message $M_2 = \langle G_2, T_2^{CAS}, Z_{UID} \rangle$ to AID.
  - *AID*: AID receives $M_2$, obtains timestamp $T_3^{AID}$, and verifies the freshness condition. If the condition is valid, AID decrypts $G_2$ to obtain the parameters $\langle AS_{ID}, sess\_id, T_2^{CAS}, n_2^{CAS}, k \rangle$. It stores the CAS identifier $AS_{ID}$ securely.
  - AID verifies the correctness of the received parameters by checking $(T_2^{CAS} == T_2^{CAS'}) \wedge (AS_{ID} == AS_{ID'})$. If true, AID further decrypts $Z_{UID}$ to retrieve $\langle A_{ID}, T_2^{CAS}, n_2^{CAS} \rangle$, and compares with its stored values. If $(T_2^{CAS} == T_2^{CAS'}) \wedge (A_{ID} == A_{ID'})$ is satisfied, AID registers the CAS.
  - After successful validation, AID computes $J = E_{sess\_id \oplus PW_D}(Z_{UID}, AS_{ID}, k)$ using XOR between $sess\_id$ and $PW_D$ as the encryption key. It stores *J* securely in its database for future use in the authentication phase.

### Authentication phase

**Fig. 5:**
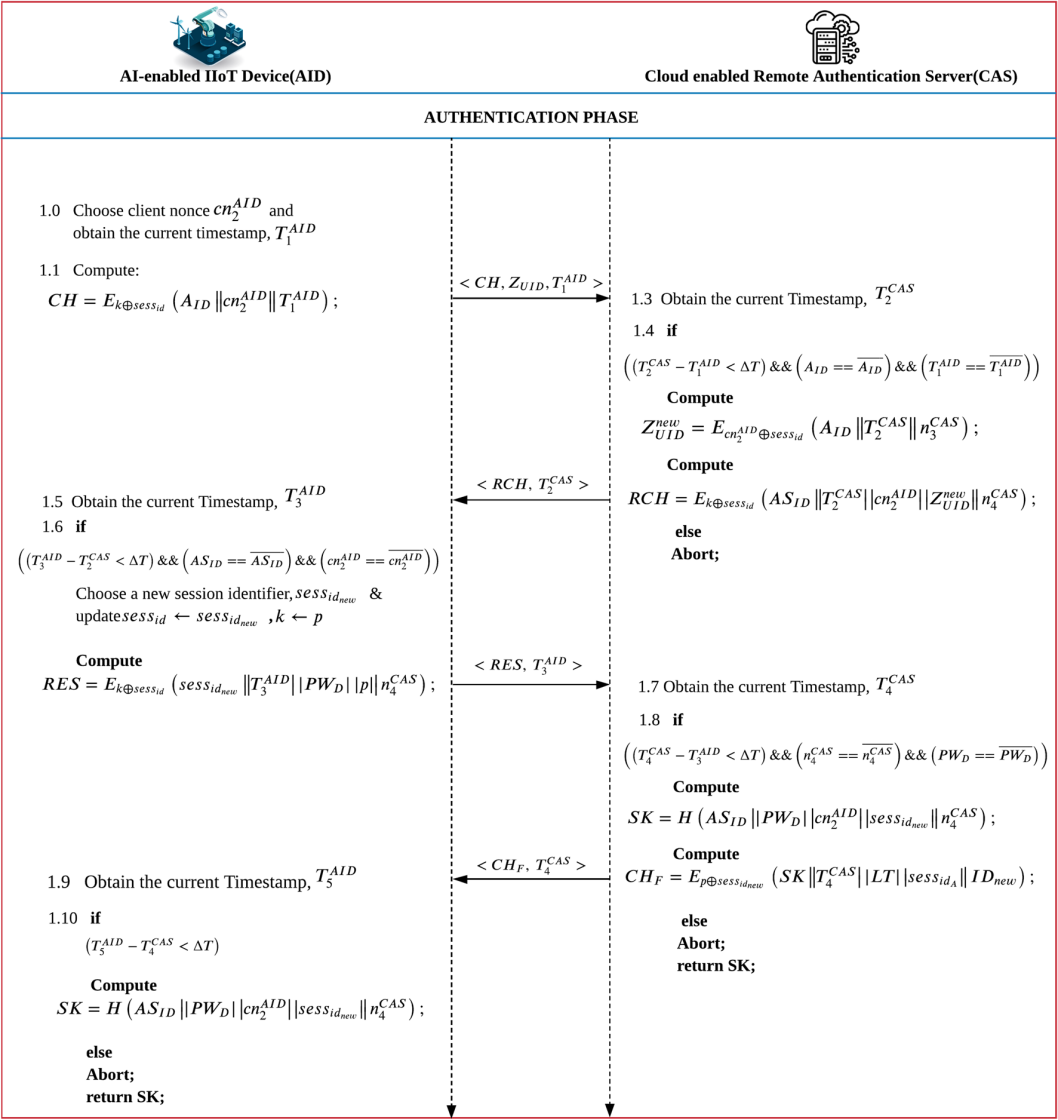
Authentication between AID and CAS.

This phase describes the mutual anonymous identity attestation process between the AID and the CAS. This procedure aims to successfully generate a secure session key between the AID and CAS, as shown in Fig. 5.

- $AID \rightarrow CAS$: When AID wishes to connect with the wireless network to invoke the network services, it decrypts the stored credentials *J*. After decrypting *J*, it obtains $\langle k, AS_{ID}, Z_{UID} \rangle$. AID then generates a pseudorandom nonce $cn_2^{AID}$ and obtains the current timestamp $T_1^{AID}$. It computes the ciphertext $CH = E_{k \oplus sess\_id}(A_{ID} \parallel cn_2^{AID} \parallel T_1^{AID})$ and sends the message $M_1 = \langle CH, T_1^{AID}, cn_2^{AID} \rangle$ to EAP.
- $EAP \rightarrow CAS$: EAP forwards the message $M_1$ to CAS.
- $CAS \rightarrow EAP$: Upon receiving $M_1$, CAS obtains a timestamp $T_2^{CAS}$ and checks the freshness condition. If valid, CAS decrypts *CH* to retrieve $\langle A_{ID}, cn_2^{AID}, T_1^{AID} \rangle$. CAS then decrypts the corresponding $Z_{UID}$ stored in its database to retrieve $\langle A_{ID}, n_2^{CAS}, sess\_id \rangle$. If the verification condition $(T_1^{AID} == T_1^{AID'}) \wedge (A_{ID} == A_{ID'})$ holds, CAS computes a new pseudonymous identifier $Z_{UID}^{new} = E_{sess\_id \oplus cn_2^{AID}}(A_{ID} \parallel n_3^{CAS} \parallel T_2^{CAS})$, where $n_3^{CAS}$ is a pseudorandom nonce generated by CAS.
- CAS then computes $RCH = E_{k \oplus sess\_id}(AS_{ID} \parallel cn_2^{AID} \parallel T_2^{CAS} \parallel Z_{UID}^{new} \parallel n_4^{CAS})$, where $n_4^{CAS}$ is another pseudorandom nonce generated by CAS. The message $M_2 = \langle RCH, T_2^{CAS} \rangle$ is sent to EAP.
- $EAP \rightarrow AID$: EAP forwards $M_2$ to AID.
- $AID \rightarrow EAP$: AID receives $M_2$ and obtains the current timestamp $T_3^{AID}$. If the freshness condition holds, it decrypts *RCH* and retrieves the parameters $\langle AS_{ID}, cn_2^{AID}, T_2^{CAS}, Z_{UID}^{new}, n_4^{CAS} \rangle$. If the condition $(cn_2^{AID} == cn_2^{AID'}) \wedge (AS_{ID} == AS_{ID'})$ is satisfied, AID confirms the legitimacy of the request and completes the authentication process.
- After successful authentication, AID selects a new session identifier $sess\_id_{new}$ and a new ephemeral key *p*. It replaces the old $sess\_id$ and *k* with the new $sess\_id_{new}$ and *p*. AID computes $RES = E_{k \oplus sess\_id}(sess\_id_{new} \parallel PW_D \parallel T_3^{AID} \parallel p \parallel n_4^{CAS})$, and sends the message $M_3 = \langle RES, T_3^{AID} \rangle$ to EAP.
- $EAP \rightarrow CAS$: EAP forwards the message $M_3$ to CAS.
- $EAP \rightarrow AID$: EAP receives message $M_5$ from CAS and extracts *LT* and $ID_{new}$. It forwards $M_5' = \langle CH_F, T_4 \rangle$ to AID.
- *AID*: Upon receiving $M_5'$ from EAP, AID obtains timestamp $T_5^{AID}$ and checks freshness. If valid, it decrypts $CH_F$ to retrieve $\langle SK, LT, T_4^{CAS}, ID_{new}, sess\_id_A \rangle$ for secure future transmissions. If this freshness check fails, this message is removed from consideration.

Rate limiting is applied to control the excessive amount of authentication requests and reduce the risk of Denial-of-Service (DoS) attacks. ZT-RIASE sets the parameter termed as *r* as the maximum number of the allocated request rate; this parameter is set at a minimum of 5 requests. When the AID or EAP exceeds this set limit, the entity is temporarily blocked/blacklisted for a fixed waiting period, and it can send the authentication request again only after this period is over. This ensures that the compromised or malicious devices cannot overwhelm the CAS with repeated login attempts and also stop the brute-force attempts, credential stuffing, and automated bot activity, while maintaining efficient authentication in the industrial 5.0 environments.

### Network failure resilient reconnection authentication (NFR)

**Fig. 6:**
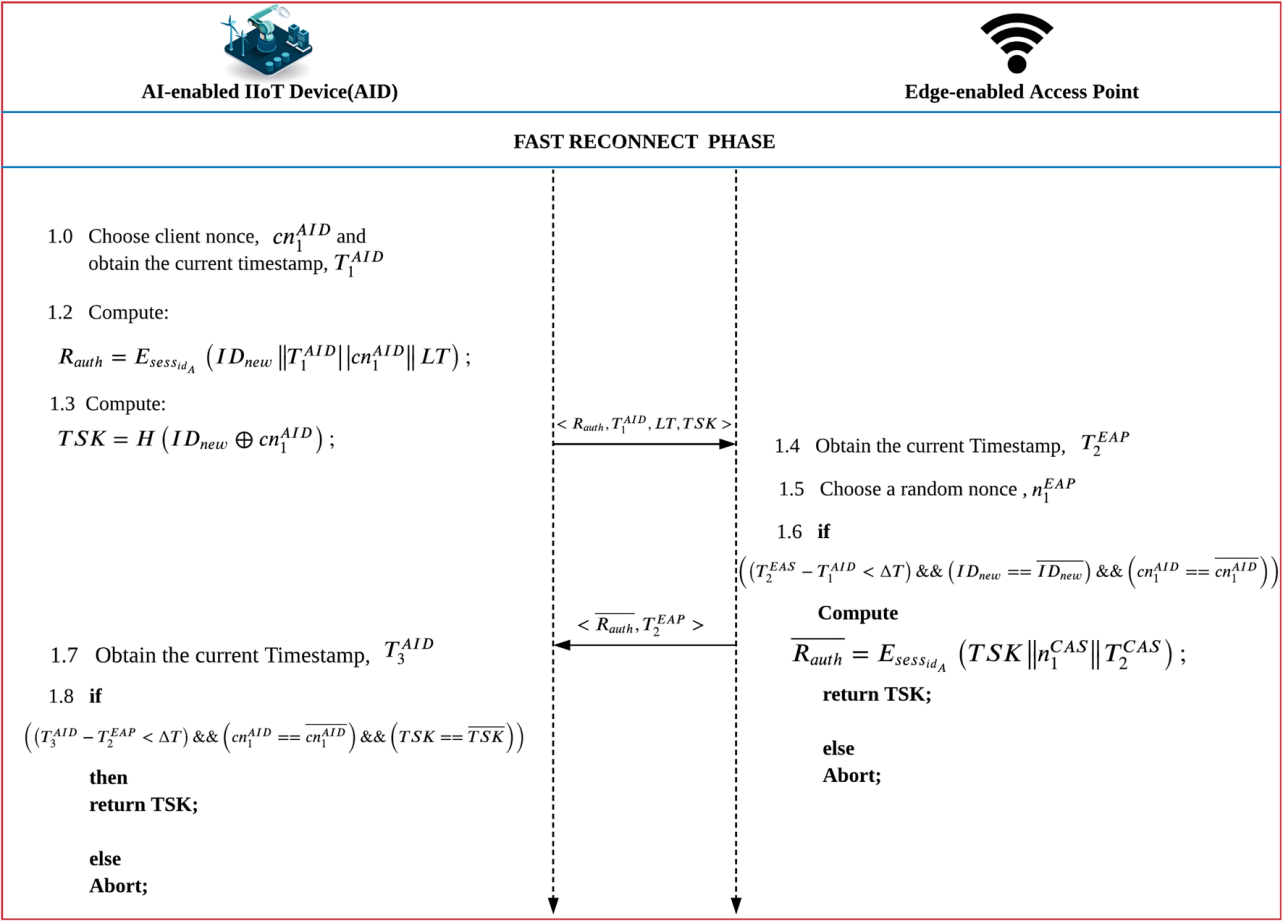
Fast Reconnect Phase/NFR phase.

In a scenario when the AID gets disassociated from the network due to some connectivity issue, network error, etc., and wishes to reconnect with the network. Then, it can quickly reconnect with the last connected EAP to rejoin the network again by simply utilizing the NFR process shown in Fig. 6 and not need to perform the whole authentication process.

- $AID \rightarrow EAP$: AID obtains the current timestamp $T_I^{AID}$ and a pseudorandom nonce $cn_1^{AID}$. It computes $R_{auth} = E_{sess\_id_A}(ID_{new} \parallel T_I^{AID} \parallel cn_1^{AID} \parallel LT)$, where $sess\_id_A$ is the session ID assigned to EAP during the registration process. AID also computes the Temporary Session Key (TSK) as $TSK = H(ID_{new} \oplus cn_1^{AID})$. This key is stored in AID’s database. Finally, AID sends the message $M_1 = \langle R_{auth}, T_I^{AID}, LT \rangle$ to EAP.
- $EAP \rightarrow AID$: Upon receiving $M_1$, EAP obtains the current timestamp $T_2^{EAP}$ and verifies the freshness condition. If the condition is satisfied, it checks whether the $(LT, ID_{new})$ pair exists in its database. If not found, the NFR request is denied. If the pair is found, EAP decrypts $R_{auth}$ to retrieve $\langle ID_{new}, T_I^{AID}, cn_1^{AID}, LT \rangle$. Then it verifies the received parameters by checking the condition: $(cn_1^{AID} == cn_1^{AID'}) \wedge (ID_{new} == ID_{new'}) \wedge (T_I^{AID} == T_I^{AID'})$. If the condition holds, EAP considers the AID’s request legitimate.
- EAP then computes $R_{auth}' = E_{sess\_id_A}(TSK \parallel n_1^{EAP} \parallel T_2^{EAP})$, where $n_1^{EAP}$ is a pseudorandom nonce generated by EAP, and $TSK = H(ID_{new} \oplus cn_1^{AID})$. EAP sends the message $M_2 = \langle R_{auth}', TSK \rangle$ to AID.
- $AID \rightarrow EAP$: Upon receiving $M_2$, AID obtains the current timestamp $T_3^{AID}$ and verifies freshness. If valid, it decrypts $R_{auth}'$ to get $\langle TSK, n_1^{EAP}, T_2^{EAP} \rangle$. AID then matches the received values with its stored values by checking: $(cn_1^{AID} == cn_1^{AID'}) \wedge (TSK == TSK') \wedge (T_2^{EAP} == T_2^{EAP'})$. If the match is successful, AID considers EAP to be legitimate.
- After successful mutual authentication, AID sends a confirmation message to EAP containing the previously sent nonce $cn_1^{AID}$, establishing the secure connection.

#### Key-derivation logic

For each successful attestation exchange, CAS and AID compute the session key as:

1 $$\begin{aligned} SK = H(cnonce_2 \parallel nonce_4 \parallel SID \parallel sess\_id^{new}) \end{aligned}$$

where $cnonce_2$ and $nonce_4$ are fresh nonces contributed by AID and CAS, respectively, and $sess\_id^{new}$ is a freshly assigned session identifier generated by CAS. The inclusion of nonces from both entities ensures bidirectional freshness, while the session identifier binds *SK* to a specific attestation context.

Reauthentication/NFR Key Refresh. Upon link failure or mobility-triggered reconnection, AID and CAS execute the two-message NFR procedure. New fresh values $(cnonce'_2, nonce'_4)$ and a new $sess\_id^{new}$ are generated, and both parties derive:

2 $$\begin{aligned} SK' = H(cnonce'_2 \parallel nonce'_4 \parallel SID \parallel sess\_id^{new}) \end{aligned}$$

thereby ensuring key independence and forward secrecy across reconnection epochs. No earlier intermediate session key is reused in $SK'$. All the protocol details, analysis, including the phase roles, key derivation, are now unified across the diagrams, BAN logic analysis, Scyther model, and performance results

### ZT integration in the ZT-RIASE framework

ZT-RIASE adopts the ZTA principles to enhance security in the framework. ZTA supports continuous verification, controlled access, and timely trust revocation. The main features of ZT are listed below:

- *Continuous Authentication and Least-Privilege Access:* ZT-RIASE enforces continuous authentication to remove the implicit trust among the communicating entities in the network. This is done by applying continuous attestation at every interaction point. Access to the resources is only provided to the entities in the required amount. RBAC and context-aware policies also adjust the required permissions when needed. If any abnormal behavior is detected in the network, then the privileges of that particular entity are revoked immediately.
- *Risk-Aware Authentication Control:* The authentication requests are evaluated using the contextual attributes such as device history and reputation. ZT-RIASE categorizes the high-risk and low-risk entities and adjusts the authentication protocol accordingly. The high-risk cases require access denial, session termination, or entity blacklisting.
- *Device and Identity Verification:* ZT-RIASE uses identity attestation to verify the identities of the AID before providing them access to the resources. The network entities, i.e., AID, EAP, and CAS, exchange the X.509 certificates to reduce the dependency on passwords and mitigate the risk of credential theft and replay attacks.
- *Micro-Segmentation and Policy Enforcement:* ZT-RIASE prevents the lateral movement by separating the network into isolated zones. This implies that an AID can communicate only with its assigned EAP, and it cannot access and communicate with any other EAP or access the data related to any segments directly.
- *Encrypted Communication:* All authentication message exchanges are safeguarded by using the AES-128 symmetric key cryptography in Galois counter mode to prevent interception and MitM attacks.
- *Threat Monitoring:* ZT-RIASE monitors authentication activities in real time to detect any unusual behaviors, patterns, or any unexpected location-based logins, or credential reuse attempts, are immediately flagged and a security action is taken.
- *Adaptive Trust Revocation:* ZT-RIASE enforces real-time trust revocation mechanisms to prevent unauthorized access, when suspicious behavior is detected, active sessions are terminated, and access is revoked.

### Network-aware crypto-behavioral continuous authentication

To reduce repeated cryptographic overhead while preserving continuous Zero Trust verification, ZT-RIASE introduces a Network-Aware Crypto-Behavioral Continuous Authentication (NA-CBCA) mechanism. In the proposed mechanism, full cryptographic identity attestation is performed during the initial session establishment between the authenticated industrial device (AID), edge access point (EAP), and central authentication server (CAS). Once the device is successfully authenticated and a valid session is created, subsequent interactions within the same session are continuously verified using lightweight behavioral authentication instead of repeatedly executing the complete cryptographic attestation protocol.

The behavioral authentication process relies on a device-specific behavior matrix constructed from session-token regularity, path/gateway consistency, command-access consistency, message-size stability, energy/processing deviation, request-frequency deviation, packet-timing deviation, and error/retransmission behavior. Since industrial network conditions may vary due to congestion, packet loss, jitter, retransmissions, and wireless interference, timing-dependent features are adjusted using a network condition index. This prevents benign network degradation from being incorrectly classified as malicious behavior. Security-critical features, such as token validity, path/gateway authorization, and command-access consistency, are not relaxed under poor network conditions because they directly indicate possible replay, session hijacking, rogue gateway access, or privilege misuse. Based on the computed behavioral risk score, the session is either continued, subjected to step-up cryptographic verification, or revoked. Thus, NA-CBCA reduces authentication delay and cryptographic cost while maintaining continuous Zero Trust enforcement.

Let $\textbf{B}_i(t)$ denote the behavioral feature vector of device $AID_i$ during monitoring window *t*:

3 $$\begin{aligned} \textbf{B}_i(t)= [ \Delta Tok_i(t), \Delta P_i(t), \Delta C_i(t), \Delta M_i(t), \Delta En_i(t), \Delta R_i(t), \Delta T_i(t), \Delta E_i(t) ], \end{aligned}$$

where $\Delta Tok_i(t)$, $\Delta P_i(t)$, $\Delta C_i(t)$, $\Delta M_i(t)$, $\Delta En_i(t)$, $\Delta R_i(t)$, $\Delta T_i(t)$, and $\Delta E_i(t)$ represent token-use deviation, path/gateway deviation, command-access deviation, message-size deviation, energy/processing deviation, request-rate deviation, packet-timing deviation, and error/retransmission deviation, respectively.

The current network condition index $\eta (t)$ is computed as:

4 $$\begin{aligned} \eta (t)= \alpha _1 \widehat{RTT}(t) + \alpha _2 \widehat{Jitter}(t) + \alpha _3 \widehat{Loss}(t) + \alpha _4 \widehat{Retx}(t), \end{aligned}$$

where $\widehat{\textrm{RTT}}(t)$, $\widehat{\textrm{Jitter}}(t)$, $\widehat{\textrm{Loss}}(t)$, and $\widehat{\textrm{Retx}}(t)$ denote the normalized round-trip time, normalized jitter, normalized packet-loss rate, and normalized retransmission rate, respectively.

The network-adjusted timing-sensitive deviations are computed as:

5 $$\begin{aligned} \Delta R_i^{adj}(t)=\frac{\Delta R_i(t)}{1+\eta (t)}, \end{aligned}$$

6 $$\begin{aligned} \Delta T_i^{adj}(t)=\frac{\Delta T_i(t)}{1+\eta (t)}, \end{aligned}$$

7 $$\begin{aligned} \Delta E_i^{adj}(t)=\frac{\Delta E_i(t)}{1+\eta (t)}. \end{aligned}$$

The behavioral risk score is then computed as:

8 $$\begin{aligned} \begin{aligned} \mathcal {R}_i^{beh}(t) =&w_1\Delta Tok_i(t) + w_2\Delta P_i(t) + w_3\Delta C_i(t) + w_4\Delta M_i(t) \\&+ w_5\Delta En_i(t) + w_6\Delta R_i^{adj}(t) + w_7\Delta T_i^{adj}(t) + w_8\Delta E_i^{adj}(t). \end{aligned} \end{aligned}$$

9 $$\begin{aligned} \mathcal {D}_i(t)= {\left\{ \begin{array}{ll} \text {Continue session}, & \mathcal {R}_i^{beh}(t)<\theta _1,\\ \text {Step-up cryptographic verification}, & \theta _1\le \mathcal {R}_i^{beh}(t)<\theta _2,\\ \text {Revoke session and alert CAS}, & \mathcal {R}_i^{beh}(t)\ge \theta _2. \end{array}\right. } \end{aligned}$$

**Algorithm 1:**
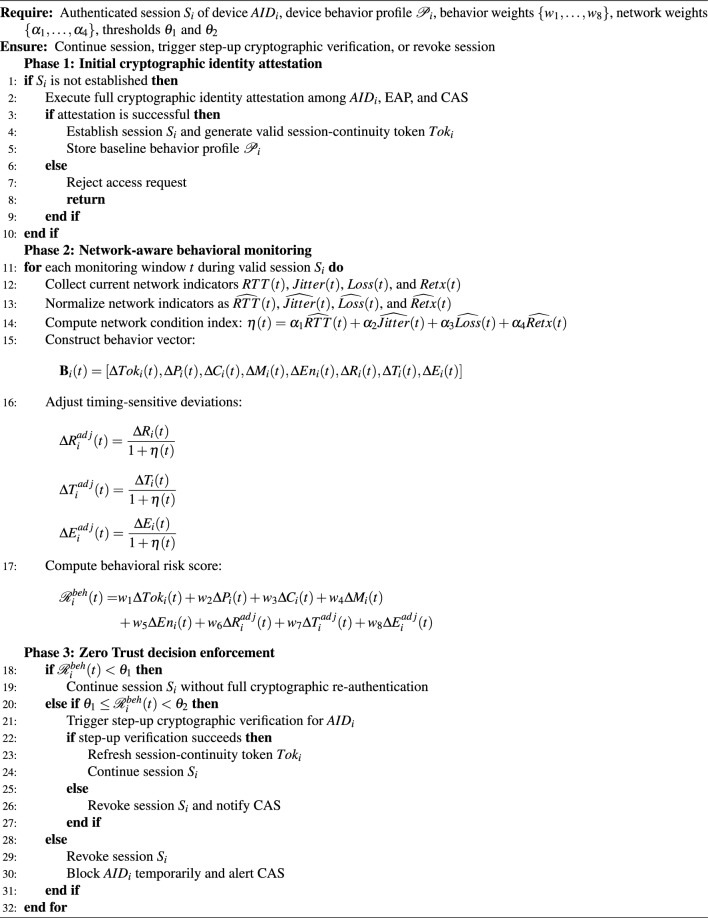
Network-Aware Crypto-Behavioral Continuous Authentication in ZT-RIASE

Algorithm 1 describes the proposed NA-CBCA mechanism. During the first interaction, the device undergoes full cryptographic identity attestation. After successful session establishment, the framework avoids repeated full cryptographic authentication and instead performs continuous behavior-based verification. The behavioral risk score strictly enforces token, path/gateway, and command-access consistency, while adapting timing, request-rate, and retransmission deviations using $\eta (t)$ to reduce false positives and decide whether to continue, step-up verify, or revoke the session.

Table 3 summarizes the phases of cryptographic usage in ZT-RIASE framework, showing that public-key cryptography is restricted to infrequent onboarding, while lightweight symmetric-key and behavior-based authentication mechanisms support session establishment, failure-resilient reconnection, and continuous Zero Trust verification during runtime.

**Table 3:** Phase-wise cryptographic usage in ZT-RIASE.

Protocol Phase	Cryptographic Basis	Purpose	Runtime Frequency
Device registration/onboarding	Public-key cryptography/key agreement	Establishes or provisions device-specific symmetric credentials securely	One-time or infrequent during commissioning
Initial identity attestation	Symmetric-key cryptography	Verifies device identity using pre-established symmetric credentials, nonce, timestamp, and message authentication	Per new session
Session establishment	Symmetric-key derivation	Derives session-specific keying material and validates freshness	Per valid session
Network-failure-resilient reconnection	Symmetric-key token verification	Enables secure session continuation after temporary link failure	Only after transient disconnection
Continuous authentication within session	Behavior-based authentication with optional symmetric step-up verification	Monitors token use, path/gateway consistency, command access, timing, and network-aware behavior	Periodic during active session

## Security analysis

### Informal security analysis

The informal security analysis of ZT-RIASE is given below:

- **Identity fraud attack:** This attack occurs when an attacker forges or misuses any identity credentials to imitate a legitimate participant of the network. ZT-RIASE safeguards against this attack by verifying the unique credentials assigned to the entity before providing access. The unique credentials: (AID’s unique ID $A_{\textrm{ID}}$, CAS’s unique ID $AS_{\textrm{ID}}$) exchanged between AID and CAS during attestation. Both entities store and cross-reference each other’s identities during authentication, ensuring secure identity attestation through database cross-referencing. AID decrypts the encrypted message and the Timestamp from CAS (i.e., $\langle RCH, T^{\textrm{CAS}}_{2} \rangle$). It also verifies the credentials $(AS_{\textrm{ID}} = AS_{\textrm{ID}}^{\prime } \ \wedge \ cn^{\textrm{AID}}_{2} = cn^{\textrm{AID}\,\prime }_{2})$, ensuring that CAS is authentic if these credentials match or else the authentication process is aborted. Similarly, when CAS receives the encrypted message $\langle RES, T^{\textrm{AID}\,\prime }_{3} \rangle$ from AID, it decrypts the message and verifies the credentials $(PW_{D} = PW_{D}^{\prime } \ \wedge \ n^{\textrm{CAS}}_{4} = n^{\textrm{CAS}\,\prime }_{4})$, ensuring authenticity of AID. The proposed scheme enhances security through the utilization of hashing, adding a layer of protection.
- **Traceable attack:** It is a privacy-compromising threat where an attacker links pseudonymous communications across sessions to reveal or infer user identities. The proposed framework achieves traceable attack protection by exchanging masked encrypted identities (the encrypted identifier of the AID as $Z_{UID}$), preventing disclosure of real identities in messages and providing protection against traceable attacks. The encrypted identifier of AID is modified after each successful authentication session (as $Z_{UID}^{new}$), preventing a correlation in messages across different sessions and enhancing security.
- **Ephemeral secret leakage (ESL) attack:** It is a cryptanalytic threat where the adversary attempts to derive session keys by exploiting transient, session-specific secrets such as nonces. The proposed framework is resilient against ephemeral secret leakage (ESL) attacks as it derives the session key from a combination of ephemeral secrets which are pseudorandom nonces $cn_2^{AID}$ and $n_4^{CAS}$ and long-term credentials $PW_D$ (i.e. password of the AID) and $AS_{ID}$ (i.e. the unique identifier of CAS), making it computationally impossible for an attacker to obtain both. The session key is derived by both the entities (i.e., AID and CAS).
- **Replay Protection:** ZT-RIASE combines timestamps and one-time nonces to prevent replay of any of the four attestation messages. In the ERP phase, AID generates a fresh challenge $cnonce_2$ and timestamp $T_1$ and sends $\{T_1, cnonce_2\}_{k}$ to CAS (message $M_1$). CAS accepts $M_1$ only if (i) $T_1$ lies within an acceptance window $\Delta T$ of its local clock and (ii) $cnonce_2$ does not appear in its table of recently used nonces. Similarly, CAS sends a fresh $nonce_4$ and timestamp $T_2$ in $M_2$, and AID accepts $M_2$ only when $T_2$ is fresh, and $nonce_4$ has not been bound to any closed session. Messages $M_3$ and $M_4$ uses the freshly generated nonces $(cnonce_2, nonce_4)$ pair along with the current timestamps $T_3$ and $T_4$. After deriving the session key *SK*,both the entities mark these nonces as used in their records. Hence, these replayed transcripts are rejected as the nonces already exists in the records or the timestamps are no longer valid. This property is listed in the BAN logic assumptions $R_3$, $R_4$, $R_9$, and $R_{10}$, and in Scyther tool by using the fresh nonce and timestamp declarations with the Alive, Niagree, and Nisynch claims.
- **Forward and Backward Secrecy with Session-Key Independence:** ZT-RIASE derives each session key from fresh nonces and a renewed session identifier as
  10 $$\begin{aligned} SK = H(cnonce_2 \oplus nonce_4 \oplus SID \oplus PW_D \oplus sess\_id^{new}), \end{aligned}$$
  where $(cnonce_2, nonce_4)$ and $sess\_id^{new}$ are regenerated in every attestation round. Hence, session keys remain independent and unlinkable under the one-way and collision-resistant properties of $H(\cdot )$. Even if long-term credentials $\langle AP_{ID}, PW_D, AS_{ID} \rangle$ or a previous *SK* are exposed, past and future keys remain protected because nonces are not reused and $\langle k, sess\_id \rangle$ is refreshed as $\langle L, sess\_id^{new} \rangle$ after authentication.
- **Timestamp Synchronization and Freshness Window:** ZT-RIASE uses loosely synchronized AID–CAS clocks, supported by NTP/PTP in industrial systems. A received timestamp is accepted only when $|T_{recv} - T_{local}| \le \Delta T .$ Messages outside this skew window are rejected and the attestation round is aborted. With fresh nonces and monotone session identifiers, this prevents replay, delay, and reordering-based desynchronization. BAN logic captures this through freshness assumptions for $T_1$–$T_4$ ($R_3$, $R_4$, $R_9$, $R_{10}$), while Scyther models timestamps as fresh terms supporting Alive and Secret claims.
- **Key-Refresh and NFR Reconnection Logic:** ZT-RIASE enables NFR reconnection by renewing session keys and identifiers without full re-registration. After disruption or session expiry, AID and CAS generate fresh nonces $(cnonce_2', nonce_4')$ and a new $sess\_id^{new'}$ to compute
  11 $$\begin{aligned} SK' = H\!\left( cnonce_2' \oplus nonce_4' \oplus SID \oplus PW_D \oplus sess\_id^{new'}\right) . \end{aligned}$$
  The old $(SK, sess\_id)$ pair is invalidated and deleted from the active session table. Since each reconnection uses new randomness and a fresh session identifier, an exposed old *SK* cannot derive $SK'$ or support impersonation. Replayed attempts fail nonce/timestamp checks and BAN-based key-binding assumptions, while rate-limited reconnection prevents stale cryptographic-state reuse.
- **Privileged-insider attack:** A privileged insider may misuse authorized access to perform unauthorized actions. ZT-RIASE limits this risk by encrypting stored AID, EAP, and CAS credentials with $\langle PW_D, PW_A, PW_{AS} \rangle$, respectively, preventing direct disclosure or misuse even if stored records are accessed.
- **Jamming and desynchronization attacks:** Jamming and desynchronization attacks disrupt communication or protocol state. ZT-RIASE counters them using freshness, credential, and message-consistency checks. Failed checks abort attestation, while temporary copies of *k* and $sess\_id$ are retained only until completion. If no valid acknowledgement or retransmission of $CH_F$ occurs within $\Delta T$, the stored state is discarded, EAP informs CAS, and CAS removes its matching state, preventing inconsistent sessions.
- **DoS and DDoS attacks:** DoS/DDoS attacks exhaust resources through high-rate request flooding. ZT-RIASE mitigates them using rate limiting, where AIDs exceeding the request threshold are flagged for inspection. Encrypted credentials also protect stored data during attack attempts. The computation cost under attack load is
  12 $$\begin{aligned} C_{\text {comp}}(t) = \alpha (t) \cdot \sum _{x \in \{\text {CAS}, \text {EAP}, \text {AID}\}} d_x(t) \cdot \left[ \gamma _h n_h^x(t) + \gamma _{\text {sym}} n_{\text {sym}}^x(t) + \gamma _{\text {asy}} n_{\text {asy}}^x(t) \right] . \end{aligned}$$
- **Rogue Access Point (RAP) attack:** A RAP attack involves a malicious access point impersonating a trusted node. ZT-RIASE prevents this by permitting only CAS-authenticated EAPs to communicate with AIDs.
- **Man-in-the-Middle (MITM) attack:** A MITM attacker intercepts, modifies, or injects messages between communicating entities. ZT-RIASE mitigates this through encrypted communication channels, making unauthorized interception or tampering ineffective.

#### Practical attack scenario analysis

Beyond formal verification, ZT-RIASE is analyzed against practical attacks in smart industrial IoT, including replay, MITM, impersonation, DoS, session hijacking, token replay, rogue gateway access, and abnormal behavior injection. These threats are relevant because IIoT devices operate over unstable links, interact through edge access points, and require repeated identity verification under Zero Trust.

- **Replay attack:** A replay attack reuses an old authentication or reconnection message $M_i$ to gain unauthorized access. ZT-RIASE accepts a message only if $|T_{recv}-T_i| \le \Delta T,$ where $\Delta T$ is the freshness window. Each token is also bound to the session identifier and expiry time as $Tok_i = H(K_{sess,i} \parallel ID_i \parallel SID_i \parallel T_{exp}).$ Thus, stale messages or expired tokens fail freshness verification and are rejected.
- **Man-in-the-Middle (MITM) attack:** A MITM attacker attempts to intercept, modify, or inject messages between communicating entities. ZT-RIASE prevents this using AES-128-GCM protection and hash-based integrity checks. For $C_i = Enc_{K_{sess,i}}(ID_i \parallel N_i \parallel T_i \parallel SID_i),$ the receiver verifies $Tag_i {\mathop {=}\limits ^{?}} MAC_{K_{sess,i}}(C_i \parallel N_i \parallel T_i).$ Without $K_{sess,i}$, any modification produces an invalid tag, causing message rejection.
- **Impersonation attack:** An impersonation attack attempts to forge a legitimate device or edge entity. ZT-RIASE requires proof of the device-specific symmetric credential $K_i$ through $Auth_i = MAC_{K_i}(ID_i \parallel N_i \parallel T_i).$ An adversary without $K_i$ cannot generate a valid authenticator. In addition, behavioral risk is computed as $\mathcal {R}_i^{beh}(t)=\sum _{k=1}^{8} w_k \Delta b_{i,k}(t),$ and if $\mathcal {R}_i^{beh}(t)\ge \theta _1$, step-up verification is triggered to prevent sustained misuse.
- **Denial-of-Service (DoS) attack:** A DoS attack floods the edge access point or authentication server with excessive, malformed, or invalid requests. ZT-RIASE mitigates this through lightweight pre-verification before full authentication processing. A request $Req_i$ is processed only if $|T_{recv}-T_i| \le \Delta T,\quad Tok_i \in \mathcal {T}_{valid},\quad r_i(t)\le R_{max},$ where $\mathcal {T}_{valid}$ is the valid-token set, $r_i(t)$ is the request rate of device *i*, and $R_{max}$ is the maximum permitted request rate. Requests violating these conditions are dropped early, reducing unnecessary cryptographic computation at EAP and CAS.
- **Session hijacking attack:** It is a session-takeover attack where the adversary attempts to misuse an already established authenticated session. The proposed framework mitigates session hijacking by binding the session-continuity token to the device identity, session identifier, expiry time, and policy context: $Tok_i = H(K_{sess,i} \parallel ID_i \parallel SID_i \parallel T_{exp} \parallel Policy_i).$ A copied token cannot be reused from a different identity, session, or policy context. Furthermore, NA-CBCA continuously checks token-use regularity, path/gateway consistency, and command-access consistency. If $\Delta Tok_i(t)+\Delta P_i(t)+\Delta C_i(t) \ge \theta _{hijack},$ the session is marked suspicious and step-up cryptographic verification is initiated.
- **Token replay attack:** It is a reconnection-phase attack where the adversary reuses a previously valid session-continuity token after network failure or session disruption. ZT-RIASE validates token freshness using $T_{current} < T_{exp}$ and checks whether the token belongs to the active session: $SID_i^{recv} {\mathop {=}\limits ^{?}} SID_i^{active}.$ If either condition fails, the token is rejected. Repeated failed token attempts are captured through
  13 $$\begin{aligned} \Delta Tok_i(t)=\frac{N_{tok}^{fail}(t)+N_{tok}^{replay}(t)}{N_{tok}^{use}(t)+1} \end{aligned}$$
  and high token-use deviation triggers re-attestation or session revocation.
- **Rogue gateway attack:** It is an edge-level attack where an unauthorized or compromised gateway attempts to redirect, intercept, or process device communication. ZT-RIASE mitigates rogue gateway attacks using path/gateway consistency checking. For each device $AID_i$, an allowed gateway set $\mathcal {G}_i^{allow}$ is maintained. The path deviation is computed as
  14 $$\begin{aligned} \Delta P_i(t)= {\left\{ \begin{array}{ll} 0, & EAP_i(t)\in \mathcal {G}_i^{allow},\\ 1, & EAP_i(t)\notin \mathcal {G}_i^{allow}. \end{array}\right. } \end{aligned}$$
  If $\Delta P_i(t)=1$, the communication path is treated as suspicious and the session is escalated for revalidation.
- **Privilege misuse attack:** It is an access-control attack where a legitimate or compromised device attempts to execute commands beyond its assigned role or privilege level. Let $\mathcal {C}_i^{allow}$ denote the set of commands permitted for device $AID_i$. The command-access deviation is computed as
  15 $$\begin{aligned} \Delta C_i(t)= \frac{N_i^{\textrm{unauth}}(t)}{N_i^{\textrm{cmd}}(t)+1} \end{aligned}$$
  where $N_i^{unauth}(t)$ is the number of unauthorized command attempts and $N_i^{cmd}(t)$ is the total number of commands in window *t*. If $\Delta C_i(t)$ exceeds the command-risk threshold $\theta _C$, the command is denied and the behavioral risk score is increased.
- **Behavior manipulation attack:** A behavior manipulation attack gradually alters traffic, request, command, or message patterns within a valid session to evade detection. ZT-RIASE mitigates this attack using the NA-CBCA behavior matrix:
  16 $$\begin{aligned} \textbf{B}_i(t)= \left[ \Delta Tok_i(t), \Delta P_i(t), \Delta C_i(t), \Delta M_i(t), \Delta En_i(t), \Delta R_i(t), \Delta T_i(t), \Delta E_i(t) \right] \end{aligned}$$
  To avoid false positives under changing network conditions, timing-sensitive deviations are adjusted using the network condition index
  17 $$\begin{aligned} \eta (t)= \alpha _1 \widehat{RTT}(t) + \alpha _2 \widehat{Jitter}(t) + \alpha _3 \widehat{Loss}(t) + \alpha _4 \widehat{Retx}(t) \end{aligned}$$
  If $\mathcal {R}_i^{beh}(t)\ge \theta _2$, the session is revoked; if $\theta _1\le \mathcal {R}_i^{beh}(t)<\theta _2$, step-up cryptographic verification is triggered.

Table 4 summarizes the resilience of ZT-RIASE against practical attack scenarios in smart industrial IoT environments.

**Table 4:** Practical attack scenario analysis of ZT-RIASE.

Attack Scenario	Adversarial Capability	ZT-RIASE Defense Mechanism	Security Outcome
Replay attack	Captures and retransmits old authentication or reconnection messages	Nonce–timestamp freshness, validity-window checking, session identifier, and token expiry	Old messages and stale tokens are rejected.
Man-in-the-middle attack	Intercepts, modifies, or injects messages between AID, EAP, and CAS	AES-128-GCM message protection, hash-based integrity verification, and symmetric credential validation	Modified or forged transcripts fail integrity and authentication checks.
Impersonation attack	Attempts to act as a legitimate AID or edge entity	Proof of possession of device-specific symmetric credential and behavior-profile verification	Unauthorized entities cannot generate valid attestation transcripts or sustain normal behavior.
Denial-of-service attack	Floods EAP/CAS with repeated requests, malformed packets, or reconnection attempts	Timestamp filtering, invalid-token rejection, lightweight pre-verification, and rate-limited access handling	Invalid traffic is discarded early, reducing unnecessary cryptographic processing.
Session hijacking	Attempts to reuse or take over an active authenticated session	Session-continuity token bound to identity, session identifier, expiry time, and policy context	Copied or stale tokens cannot be reused outside the valid session context.
Token replay attack	Reuses a previously valid continuity token after disruption	Token expiry checking, session binding, and token-use regularity monitoring	Replayed continuity tokens are rejected and suspicious behavior triggers step-up verification.
Rogue gateway attack	Redirects communication through an unauthorized EAP or malicious gateway	Path/gateway consistency check and allowed-EAP validation in NA-CBCA	Unauthorized path changes increase risk and trigger revalidation or session blocking.
Privilege misuse	Legitimate or compromised device requests unauthorized commands	Command-access consistency checking and least-privilege enforcement	Policy-violating commands are denied and the session risk score increases.
Behavior manipulation	Gradually changes traffic, timing, command, or message behavior during a valid session	NA-CBCA behavior matrix with token, path, command, message-size, request-rate, timing, error, and energy features	Abnormal behavior is detected through behavioral risk scoring and escalated to step-up verification.

#### Formal security analysis using BAN logic

ZT-RIASE is formally analyzed using Burrows–Abadi–Needham (BAN) logic^35^ to verify mutual authentication and session-key establishment between AID and CAS. BAN logic reasons about authentication beliefs, freshness, and jurisdiction by abstracting low-level cryptographic operations. Accordingly, the adversary model, initial assumptions, idealized messages, and target security goals are defined before deriving the proof.

Adversarial Model for BAN Logic: The BAN analysis follows a standard Dolev–Yao (DY) attacker with full control over the communication channel. The adversary may intercept, replay, reorder, or synthesize messages, but cannot break cryptographic primitives, forge encrypted messages without the correct key, or guess fresh nonces or timestamps. BAN logic does not model computational hardness; rather, it assumes perfect cryptography and focuses on authentication and belief transfer.

Security Properties to be Proven: BAN logic is used here to establish the following formal goals:

- **Goal 1:** $$AS \mid \equiv D \overset{SK}{\leftrightarrow }\ AS$$
  The CAS believes that *SK* is a good shared session key with AID.
- **Goal 2:** $$D \mid \equiv D \overset{SK}{\leftrightarrow }\ AS$$
  The AID believes that *SK* is a good shared session key with CAS.

These goals capture mutual authentication and confirm that both parties agree on the same fresh session key.

Initial Beliefs and Assumptions: BAN logic requires that both parties start with certain beliefs regarding long-term keys, freshness assumptions, and jurisdiction. These are now explicitly defined as follows:

- $$D \mid \equiv D \overset{k}{\leftrightarrow }\ AS$$
  AID believes that long-term key *k* is securely shared with CAS.
- $$AS \mid \equiv D \overset{k}{\leftrightarrow }\ AS$$
  CAS believes the same about *k*.
- $$D \mid \equiv \#T_2, \#T_3$$ and $$AS \mid \equiv \#T_1, \#T_4$$
  Both parties believe timestamps generated by themselves are fresh.
- $$AS \mid \equiv D \Rightarrow p$$
  CAS believes AID has jurisdiction over the value *p* (AID’s proof token).
- $$AS \mid \equiv D \Rightarrow sess\_id^{new}$$
  CAS believes AID can speak for the new session identifier.
- $$D \mid \equiv AS \Rightarrow (D \overset{SK}{\leftrightarrow }\ AS)$$
  AID believes CAS is authoritative for establishing session keys.

These are consistent with secure real-world deployments where long-term keys are pre-shared during device initialization.

Message Idealization: Real protocol messages contain AES encryptions, XOR operations, and concatenated metadata. BAN logic abstracts away cryptographic structure and models only the beliefs conveyed. The idealized messages below reflect the semantic information transmitted while omitting cryptographic details.

- $$M_1$$: $$D \rightarrow AS: (HID \parallel T_1 \parallel cnonce_1)$$
- $$M_2$$: $$AS \rightarrow D: (SID \parallel T_2 \parallel nonce_4 \parallel cnonce_2)_{k \oplus sess\_id}$$
- $$M_3$$: $$D \rightarrow AS: (PW_D \parallel T_3 \parallel nonce_4 \parallel AS \mid \overset{p}{\leftrightarrow }\ D \parallel AS \mid D)_{k \oplus sess\_id}$$
- $$M_4$$: $$AS \rightarrow D: (LT \parallel ID_{new} \parallel T_4 \parallel D \overset{SK}{\leftrightarrow }\ AS \parallel sess\_id_A)_{p \oplus sess\_id^{new}}$$

Notation and Rules: Tables 5 and 6 list the BAN logic symbols and rules used for the derivation. These tables were expanded to include freshness assumptions, key-binding rules, and relevant jurisdiction statements.

**Table 5:** BAN Logic Notation and Symbols Used in ZT-RIASE Analysis.

Notation	Meaning
$P \mid \equiv X$	Principal *P* believes statement *X*
$P \mid \sim X$	Principal *P* once said *X*
$P \mid \Rightarrow X$	Principal *P* has jurisdiction over *X*
$\#X$	Statement *X* is fresh
$P \overset{K}{\leftrightarrow }\ Q$	*P* and *Q* share a good secret key *K*
$\{X\}_K$	Message *X* encrypted using key *K*
*HID*	Hidden (pseudo) identity of AID
*SID*	Session identifier
*SK*	Established session key
*k*	Long-term shared key between AID and CAS
$T_i$	Timestamp generated in protocol step *i*
$nonce_i$	Random nonce generated in protocol step *i*
$cnonce_i$	Challenge nonce used for freshness proof
*p*	AID-issued proof token
$sess\_id^{new}$	Newly generated session identifier

**Table 6:** BAN Logic Rules Applied in ZT-RIASE Security Proof.

Rule ID	Rule Description
$R_1$ (Message Meaning)	If $P \mid \equiv P \overset{K}{\leftrightarrow }\ Q$ and *P* sees $\{X\}_K$, then $P \mid \equiv Q \mid \sim X$
$R_3$ (Nonce Verification)	If $P \mid \equiv \#X$and$P \mid \equiv Q \mid \sim X$, then $P \mid \equiv Q \mid \equiv X$
$R_4$ (Freshness Rule)	If $P \mid \equiv \#(X,Y)$, then $P \mid \equiv \#X$
$R_5$ (Seeing Rule)	If *P* receives *X*, then *P* sees *X*
$R_6$ (Decomposition Rule)	If *P* sees (*X*, *Y*), then *P* sees *X* and *Y*
$R_7$ (Message Meaning with XOR-bound key)	If $P \mid \equiv P \overset{K \oplus S}{\leftrightarrow }\ Q$ and *P* sees $\{X\}_{K \oplus S}$, then $P \mid \equiv Q \mid \sim X$
$R_9$ (Timestamp Verification)	If $P \mid \equiv \#T$ and $P \mid \equiv Q \mid \sim (X,T)$, then $P \mid \equiv Q \mid \equiv X$
$R_{10}$ (Extended Nonce Rule)	If $P \mid \equiv \#(X,Y)$ and $P \mid \equiv Q \mid \sim (X,Y)$, then $P \mid \equiv Q \mid \equiv (X,Y)$
$R_{11}$ (Jurisdiction Rule — Proof Token)	If $P \mid \equiv Q \Rightarrow X$ and $P \mid \equiv Q \mid \equiv X$, then $P \mid \equiv X$
$R_{12}$ (Jurisdiction Rule — Session Identifier)	If $P \mid \equiv Q \Rightarrow sess\_id^{new}$ and $P \mid \equiv Q \mid \equiv sess\_id^{new}$, then $P \mid \equiv sess\_id^{new}$
$R_{13}$ (Session Key Acceptance)	If $P \mid \equiv Q \mid \equiv P \overset{SK}{\leftrightarrow }\ Q$, then $P \mid \equiv P \overset{SK}{\leftrightarrow }\ Q$
$R_{15}$ (Key Confirmation Rule)	If $P \mid \equiv Q \overset{SK}{\leftrightarrow }\ P$ and $P \mid \equiv \#SK$, then $P \mid \equiv P \overset{SK}{\leftrightarrow }\ Q$

Derivation of Security Goals:

- **Step S1**: Using $R_7$ and the MM rule on $M_1$, $AS \mid \equiv D \mid \sim M_1$
- **Step S2**: Using $R_9$ and the TV rule on $S_1$, $AS \mid \equiv D \mid \equiv (HID, cnonce_1)$
- **Step S3**: Using $R_1$ and MM rule on $M_2$, $D \mid \equiv AS \mid \sim M_2$
- **Step S4**: Using $R_3$ and TV rule on $S_3$, $D \mid \equiv AS \mid \equiv (SID, nonce_4, cnonce_2)$
- **Step S5**: Using $R_7$ and MM rule on $M_3$, $AS \mid \equiv D \mid \sim M_3$
- **Step S6**: Using $R_{10}$ and TV rule on $S_5$, $AS \mid \equiv D \mid \equiv (PW_D, nonce_4, sess\_id^{new}, p)$
- **Step S7**: JR rule from $S_6$ and $R_{11}$, $AS \mid \equiv D \overset{p}{\leftrightarrow }\ AS$
- **Step S8**: JR rule from $S_6$ and $R_{12}$, $AS \mid \equiv D \overset{sess\_id^{new}}{\leftrightarrow } AS$
- **Step S9**: Combining $S_2$, $S_6$, $S_8$, and the SK definition, $AS \mid \equiv D \mid \equiv D \overset{SK}{\leftrightarrow }\ AS$
- **Step S10**: JR rule from $S_9$ and $R_{15}$, $AS \mid \equiv D \overset{SK}{\leftrightarrow }\ AS$ **(Goal 1)**
- **Step S11**: MM rule from $R_5$, $R_6$ on $M_4$, $D \mid \equiv AS \mid \sim M_4$
- **Step S12**: TV rule from $S_{11}$ and $R_4$, $D \mid \equiv AS \mid \equiv (LT, ID_{new}, sess\_id_A, D \overset{SK}{\leftrightarrow }\ AS)$
- **Step S13**: JR rule from $S_{12}$ and $R_{13}$, $D \mid \equiv D \overset{SK}{\leftrightarrow }\ AS$ **(Goal 2)**

Given the assumptions, message idealization, and rule-based derivation, both AID and CAS believe they share the fresh session key *SK*. Thus, the ZT-RIASE protocol achieves mutual authentication and secure key establishment under BAN logic.

#### Formal security analysis using the Scyther tool

**Fig. 7:**
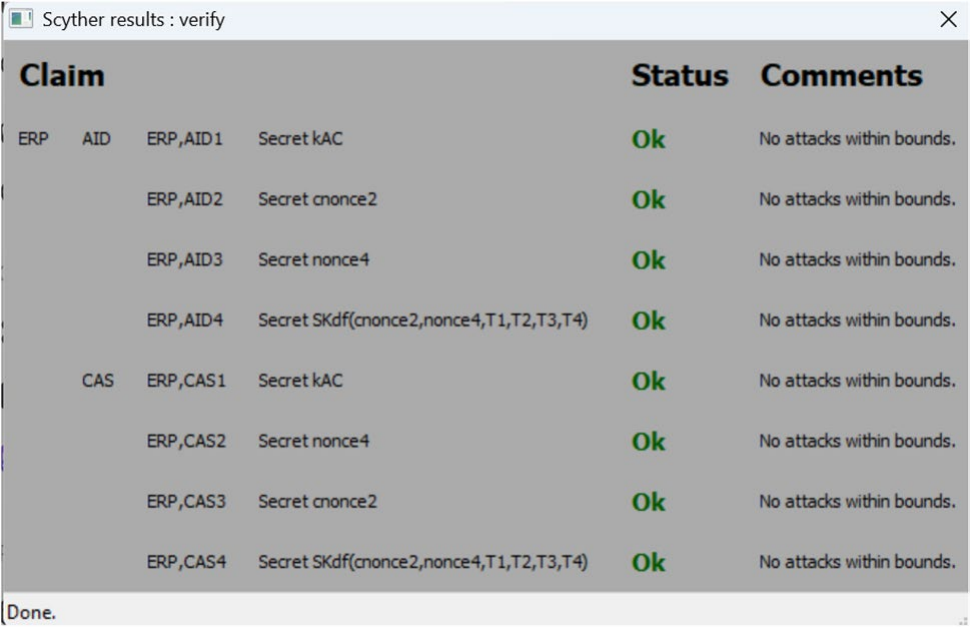
Scyther Tool Analysis.

The research paper employs the Scyther^36^, a formal verification tool, to assess the security of the proposed framework. Security frameworks are evaluated using the Security Framework Description Language (.spdl). The results illustrate that ZT-RIASE satisfies all the defined security claims, which include event execution, message delivery, and maintaining the secrecy. The reported analysis results are shown in Fig. 7. The detailed analysis specification of ZT-RIASE is given in Appendix A.

## Performance analysis

This section provides the performance results of ZT-RIASE. It gives the computation, communication, storage, and energy costs analysis results to assess the overall efficiency. All the unified descriptions of the hardware and the simulation platforms used in the evaluation of the ZT-RIASE. Table 7 gives the exact platform specifications, and Table 8 summarizes the abbreviations used in the performance analysis.

**Device-side platform (AID):** All cryptographic runtimes for AES-128, SHA-256, nonce generation, and attestation-payload construction were measured on a Raspberry Pi 4B equipped with a quad-core Cortex-A72 CPU (4$\times$1.5 GHz) and 4 GB RAM, running Raspberry Pi OS 64-bit. Earlier inconsistencies in the description of the Raspberry Pi model and configuration have been removed; the revised manuscript consistently refers to this platform as the AID device.

**Microcontroller Baseline:** Microcontroller-level energy baselines were obtained from an Arduino Uno board based on the ATmega328P (16 MHz, 2 KB SRAM) programmed via the Arduino IDE runtime. These measurements are used only as a reference for ultra-constrained IIoT nodes and do not overlap with Raspberry Pi timing results.

**Desktop Testbed (CAS and simulation host):** CAS-side verification latency and all simulation traces were generated on a desktop testbed with a 12th Gen Intel Core i7-12700K processor (3.60 GHz), 16 GB RAM, and Windows 11 Pro (Version 22H2). The previous version of the manuscript listed this platform only as a “desktop testbed” without CPU, RAM, or OS information; Table 7 now provides complete specifications.

**Network Simulator:** All network-level metrics—including authentication delay, RTT, reconnection time, and energy consumption under varying loads—were obtained using the ns-3.41 simulator. We correct the earlier mislabeling of the simulator: OMNeT++ was not used in any experiment. The PHY and MAC layers were modelled using the YansWifiPhy, WifiMacQueue, and BasicEnergySource modules, which explain the ns-3-style energy and PHY behaviour observed in the results.

To avoid ambiguity, each result is explicitly tied to its originating platform. Cryptographic computation and communication costs in Tables 9 and 10 originate from the Raspberry Pi 4B, microcontroller energy baselines in Table 11 are from the Arduino Uno, while authentication delay, RTT, throughput, and reconnection latency in Figures 10,11,12,13,14,15 are derived solely from ns-3.41 simulations executed on the desktop testbed.

**Table 7:** Experimental hardware and simulation platforms used in evaluation.

Platform	CPU	RAM	Operating system	Primary usage
Raspberry Pi 4B	ARM Cortex–A72 (4 $\times$ 1.5 GHz)	4 GB	Raspberry Pi OS (64-bit)	Device-side cryptographic operations and attestation latency
Arduino Uno	ATmega328P (16 MHz)	2 KB	Arduino runtime	Microcontroller-level energy consumption baseline
Desktop testbed	Intel Core i7–12700K (3.60 GHz)	16 GB	Windows 11 Pro (22H2)	CAS-side verification and *ns-3.41* simulation hosting
Network simulator	*ns-3.41*	–	–	Network latency, RTT, reconnection delay, and energy modeling

**Table 8:** Performance Parameters Abbreviations.

Symbol	Description
$C_{\text {comp}}$	Total computation cost per authentication cycle (in milliseconds)
$N_{\text {op}_i}$	Number of cryptographic operations of type *i*
$T_{\text {op}_i}$	Execution time for cryptographic operation of type *i* (in ms)
$N_{\text {hash}}$	Number of hash operations
$T_{\text {hash}}$	Time per hash operation
$N_{\text {aes}}$	Number of AES operations
$T_{\text {aes}}$	Time per AES operation
$N_{\text {rsa}}$	Number of RSA operations
$T_{\text {rsa}}$	Time per RSA operation
$N_{\text {ecc}}$	Number of ECC operations
$T_{\text {ecc}}$	Time per ECC operation
$N_{\text {dh}}$	Number of Diffie-Hellman operations
$T_{\text {dh}}$	Time per Diffie-Hellman operation
$C_{\text {comm}}$	Total communication cost (in bits)
$N_{\text {msg}_i}$	Number of messages of type *i*
$L_{\text {msg}_j}$	Bit-length of each message of type *j*
$C_{\text {store}}$	Total storage cost (in bits)
$N_{\text {key}}$	Number of cryptographic keys stored
$S_{\text {key}}$	Size of each stored key (in bits)
$N_{\text {state}}$	Number of stored session/context states
$S_{\text {state}}$	Size of each session/context state (in bits)
$C_{\text {energy}}$	Total energy cost per authentication cycle (in millijoules)
$E_{\text {tx}}$	Energy consumed for transmitting 1 bit
$E_{\text {aes}}$	Energy consumed per AES operation
$E_{\text {h}}$	Energy consumed per hash operation
$E_{\text {det}}$	Energy consumed for anomaly detection per node
$R_{\text {extra}}$	Number of retransmissions due to malicious interference
$D_{\text {extra}}$	Number of additional anomaly detection cycles triggered
$N_{\text {bits}}$	Number of bits exchanged
$\alpha$	Interference rate due to malicious nodes ($0 \le \alpha \le 1$)
*NMR*	Node Misbehavior Rate
$N_{\text {mis}}$	Number of misbehaved actions or events
$N_{\text {total}}$	Total number of observed actions
$w_i$	Severity weight of misbehavior instance *i*
*N*	Number of total actions
$M \subseteq \{1,2,\dots ,N\}$	Indices of misbehaving actions
*CNDR*	Compromised Node Detection Rate
$N_{\text {cd}}$	Number of correctly detected compromised nodes
$N_{\text {ct}}$	Total number of actual compromised nodes
$N_c(t)$	Set of compromised nodes at time *t*
$N_{\text {cd}}(t)$	Set of compromised nodes correctly detected at time *t*
*T*	Total observation time window
$T_{\theta }$	Dynamic trust threshold used to flag a node as compromised
$P_d(n,t)$	Probability of detecting node *n* as compromised at time *t*
$\omega _t$	Weighting function to emphasize recent detections (e.g., exponential decay)
$\alpha$	Penalty term for false alarms or false positives
*FPR*	False Positive Rate
$N_{\text {fp}}$	Benign nodes wrongly flagged as compromised
$N_{\text {bn}}$	Total number of actual benign nodes
$N_{\text {tn}}$	Benign nodes correctly classified as non-compromised
$E_{\text {auth}_{\text {base}}}$	Base energy consumption (in mJ)
$E_{\text {auth}_{\text {mal}}}$	Malicious node impact on energy (in mJ)
$E_{\text {auth}_{\text {total}}}$	Total energy consumption in presence of malicious nodes (in mJ)
*T*	Throughput (bits/sec or bps)
$D_{\text {recv}}$	Total data successfully received (in bits)
$T_{\text {total}}$	Total time required to transmit the data (in seconds)
$N_r$	Number of retransmitted packets
$O_{\text {sec}}$	Cryptographic overhead time (hash, AES, etc.)
$T_{\text {proc}}$	Processing delay per packet
$T_{\text {net}}$	Network transmission delay
*PLR*	Packet Loss Rate
$N_{\text {sent}}$	Total number of packets sent
$N_{\text {recv}}$	Total number of packets received successfully
$N_{\text {lost}}$	Total number of packets lost ($N_{\text {sent}} - N_{\text {recv}}$)

### Computation cost

**Fig. 8:**
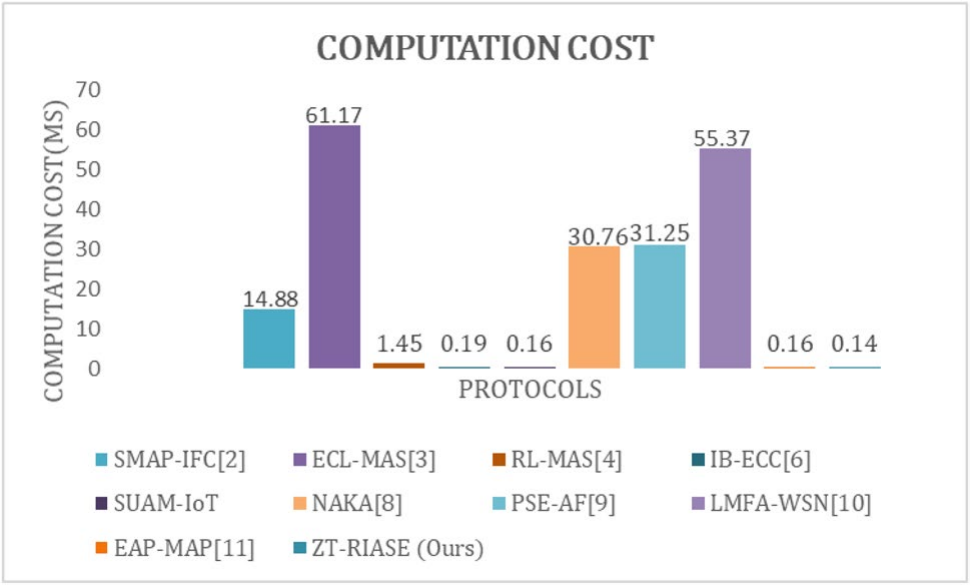
Computation Cost Analysis.

This section provides the analysis of the computation cost of the cryptographic operations in ZT-RIASE and its comparison with the state-of-the-art protocols given in Table 9. This evaluation is done by using the cypto++ library. It includes the execution time of the main cryptographic operation such as symmetric encryption, public-key algorithms, hashing, and digital signatures. The execution time of one-way hashing ($T_H$), AES-128 encryption and decryption ($T_{\text {AES}}$), RSA-2048 operations ($T_{\text {RSA}}$), Diffie–Hellman key exchange ($T_{\text {DH}}$), and elliptic-curve point multiplication ($T_{\text {PM}}$) is measured with crypto++ library given in EAP-MAP^9^,. The results are shown in Fig. 8 for the computation cost analysis. The computation cost of the ZT-RIASE is approximately 0.14 ms, expressed as $(5T_{\text {AES}} + 2T_H)$. When compared with the EAP-MAP^9^, which incurs 0.16 ms. These improvements occur because of the reduction in the number of cryptographic operations. The protocols that use asymmetric primitives, including ECC-based approaches such as ECL-MAS^13^ and IB-ECC^18^, as well as hybrid designs like NAKA^3^ and PSE-AF^2^, incur high computation cost due to point multiplications and PUF processing. Multi-round and multi-factor protocols, such as LMFA-WSN^15^ and SMAP-IFC^11^, also incur higher delays because of additional hashing, storage access, and sensor-side processing.

**Table 9:** Computation Cost Comparison.

Protocol	Computation Cost	Cost (ms)	Gain (%)
SMAP-IFC^11^	$23T_{h} + 14T_{emo} + 1T_{eao} + 4T_{se\_d}$	14.88	99.06
ECL-MAS^13^	$16T_{h} + 3T_{m}$	61.17	99.77
RL-MAS^12^	$2T_{h} + 3T_{aed} + 2T_{ES} + 2T_{CHM}$	1.45	90.34
IB-ECC^18^	$66T_{m} + 2T_{h} + 2T_{a}$	0.19	26.32
SUAM-IoT^37^	$7T_{h} + 25T_{xor} + 1T_{e} + 1T_{d}$	0.16	12.50
NAKA^3^	$6T_{D/E} + 12T_{H} + 4T_{PUF}$	30.76	99.54
PSE-AF^2^	$2T_{P} + 6T_{E} + 21T_{H}$	31.25	99.55
LMFA-WSN^15^	$18T_{h} + 1T_{f} + 7T_{sm}$	55.37	99.75
EAP-MAP^9^	$6T_{AES} + 2T_{H}$	0.16	12.50
**ZT-RIASE (Ours)**	$5T_{AES} + 2T_{H}$	**0.14**	**–**

**Note:** Gain is computed relative to ZT-RIASE as $\left( \frac{C_i - C_{\text {ZT-RIASE}}}{C_i}\right) \times 100$.

The total computation cost per authentication cycle can be calculated by using the formula:

18 $$\begin{aligned} C_{\text {comp}} = \sum _{i=1}^{n} \left( N_{\text {op}_i} \cdot T_{\text {op}_i} \right) \end{aligned}$$

Expansion from the equation (18),

19 $$\begin{aligned} C_{\text {comp}} = N_{\text {hash}} \cdot T_{\text {hash}} + N_{\text {aes}} \cdot T_{\text {aes}} + N_{\text {rsa}} \cdot T_{\text {rsa}} + N_{\text {ecc}} \cdot T_{\text {ecc}} + N_{\text {dh}} \cdot T_{\text {dh}} \end{aligned}$$

This formulation in equation (19) comprehensively models the total computational burden by aggregating all cryptographic operations invoked during a single identity attestation cycle. Each component $N_{\text {op}_i}$ is protocol-dependent and reflects the operational intensity, while $T_{\text {op}_i}$ is derived from benchmarked execution times on embedded processors using optimized cryptographic libraries.

### Communication cost

This section provides the communication cost of ZT-RIASE and its comparison with the state-of-the-art. The total communication cost is computed using the parameters given in the research work^9^. These include the identity value, random numbers generated by Psuedo Random Number Generator(PRNG), and other cryptographic elements transmitted during the attestation. Each identity and random value is assumed to be 160 bits. The sizes of cryptographic elements are set as follows: AES encryption and decryption use 128 bits, hash outputs use 256 bits, and RSA-based public-key operations use 2048 bits. For the proposed framework, the total communication overhead is calculated as $((CH, T_1, Z\_UID),(RCH, T_2),(RES, T_3),(\text {CH}_F, T_4))$, which results in approximately 768 bits per authentication session. The results are shown in Table 10, ZT-RIASE attains a reduction of 14.29% compared to its closest baseline EAP-MAP protocol^9^. The reduction is achieved due to the use of AES-128 in Galois counter mode, and limiting the number of cryptographic operations, reducing the size of the nonces and random numbers without compromising the security. The communication cost of ZT-RIASE is calculated as:

20 $$\begin{aligned} C_{\text {comm}} = \sum _{j=1}^{m} \left( N_{\text {msg}_j} \cdot L_{\text {msg}_j} \right) \end{aligned}$$

**Fig. 9:**
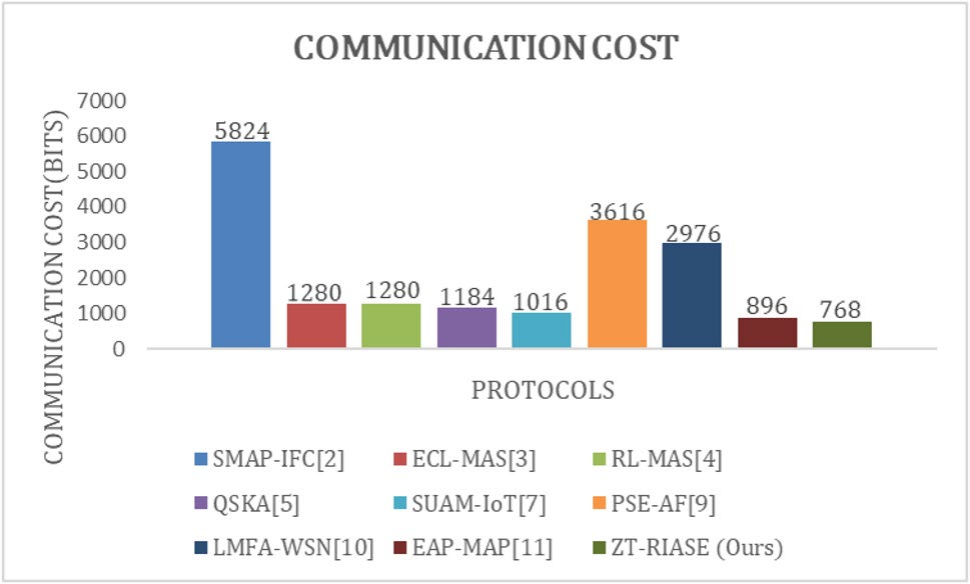
Communication Cost Analysis.

Figure 9 compares the communication cost of ZT-RIASE with its counterparts. The protocols SMAP-IFC^11^, PSE-AF^2^, and LMFA-WSN^15^ incur high overhead because of certificate-based signalling or multifactor data transfer. Also the lightweight protocols, ECL-MAS^13^, RL-MAS^12^, and QSKA^20^, adds extra overhead due to the asymmetric operations. Comparatively, ZT-RIASE incurrs cost of 768 bits, resulting in a 14.29% reduction with EAP-MAP^9^. The results show that ZT-RIASE achieves the lowest communication cost among the evaluated protocols.

**Table 10:** Communication cost comparison.

Protocol	Communication Cost (bits)	Gain over ZT-RIASE (%)
SMAP-IFC^11^	5824	86.81
ECL-MAS^13^	1280	40.00
RL-MAS^12^	1280	40.00
QSKA^20^	1184	35.14
SUAM-IoT^37^	1016	24.41
PSE-AF^2^	3616	78.76
LMFA-WSN^15^	2976	74.19
EAP-MAP^9^	896	14.29
**ZT-RIASE (Ours)**	**768**	**-**

### Storage cost

The storage cost evaluation considers the permanent data maintained by an IIoT device within the ZT-RIASE framework. Unlike EAP-based authentication schemes that require storage of multiple cryptographic parameters and credentials, ZT-RIASE stores only a single long-term symmetric key *j* and a minimal session identifier, resulting in a persistent storage requirement of 128 bits. The storage requirement per entity in ZT-RIASE is modeled as:

21 $$\begin{aligned} C_{\text {store}} = N_{\text {key}} \cdot S_{\text {key}} + N_{\text {state}} \cdot S_{\text {state}} \end{aligned}$$

ZT-RIASE reduces storage overhead by maintaining only the essential session and identity-related information per device. Specifically, symmetric session keys (e.g., AES-128), ephemeral nonces, and anonymized identifiers are cached only for active sessions as given in equation (21). Since the protocol avoids persistent or redundant state storage and employs ephemeral key regeneration, the total storage cost is constrained to 128 bits, ensuring scalability across low-memory IIoT endpoints. The analysis of storage costs demonstrates that the proposed framework exhibits lower storage requirements, making it well-suited for resource-constrained IIoT devices with limited memory capacity.

### Energy consumption

This section depicts an energy consumption analysis of the proposed framework, which is computed based on the data provided in the scheme^9^, which includes measurements for various tasks such as transmitting a bit (i.e., 0.00066 mJ), AES symmetric encryption/decryption (i.e., 0.00217 mJ), hashed output (i.e., 0.000108 mJ), and public key encryption/decryption using RSA (i.e., 15.6 mJ). For the proposed framework, the energy consumption is estimated to be approximately 0.50 mJ ($768 \times 0.00066 + 5 \times 0.000207 + 2 \times 0.000108$), considering factors such as the number of bits transmitted and the energy requirements for AES and hash function operations as shown in Table 11. ZT-RIASE’s energy cost per authentication session is modeled by aggregating transmission, cryptographic processing, and hashing costs:

22 $$\begin{aligned} C_{\text {energy}} = E_{\text {tx}} + E_{\text {crypto}} + E_{\text {hash}} \end{aligned}$$

**Table 11:** Energy baseline of ZT-RIASE per authentication session (total = 0.50).

Component (per session)	Source	Energy	Share (%)
AES-128 encryption/decryption (all invocations)	Device-side crypto	0.093	18.6
SHA-256 hashing (all invocations)	Device-side crypto	0.042	8.4
Nonce and timestamp processing	Device-side crypto	0.016	3.1
Session-key derivation (SKdf)	Device-side crypto	0.025	4.9
Wireless transmission (protocol messages)	Link-layer radio cost	0.217	43.4
Wireless reception (protocol messages)	Link-layer radio cost	0.087	17.3
**Total energy per ZT-RIASE session**	–	**0.500**	**100**

Expanded from equation (22):

23 $$\begin{aligned} C_{\text {energy}} = (N_b \cdot E_b) + (N_{\text {aes}} \cdot E_{\text {aes}}) + (N_h \cdot E_h) \end{aligned}$$

### Lightweight overhead analysis on constrained devices

To substantiate the lightweight nature of ZT-RIASE, we evaluate the runtime overhead of the proposed framework in terms of computation cost, communication overhead, memory/storage requirement, and energy consumption. Since ZT-RIASE follows a hybrid bootstrap–symmetric runtime design, the one-time public-key-based registration and key-agreement cost is reported separately from the recurring runtime cost. This distinction is important because device registration is performed only during onboarding or commissioning, whereas identity attestation, reconnection, and continuous authentication are repeatedly executed during normal IIoT operation. After the registration phase, ZT-RIASE relies on AES-128-GCM, hash-based integrity verification, nonce–timestamp freshness, symmetric session-continuity tokens, and Network-Aware Crypto-Behavioral Continuous Authentication (NA-CBCA). Therefore, the recurring runtime cost is dominated by lightweight symmetric-key operations and behavior-score computation rather than repeated public-key cryptography. The lightweight claim is supported using four quantitative indicators: computation time, communication size, memory footprint, and energy consumption on constrained or representative IIoT devices.

Let $C_{reg}$ denote the one-time public-key registration cost, $C_{sym}$ denote the symmetric runtime attestation cost, $C_{rec}$ denote the reconnection cost, and $C_{beh}$ denote the behavior-based continuous authentication cost. For $N_s$ session interactions and $N_{step}$ step-up verifications, the total runtime cost of ZT-RIASE is expressed as

24 $$\begin{aligned} C_{total}^{ZT\text {-}RIASE} = C_{reg} + C_{sym} + C_{rec} + (N_s-1)C_{beh} + N_{step}C_{sym}. \end{aligned}$$

In contrast, a repeated cryptographic authentication design that performs full cryptographic verification at every interaction incurs $C_{\textrm{total}}^{\textrm{crypto}} = C_{\textrm{reg}} + N_s C_{\textrm{crypto}}$ Since the proposed continuous authentication phase uses lightweight behavioral scoring, $C_{\textrm{beh}} \ll C_{\textrm{sym}} \ll C_{\textrm{crypto}}$ the recurring runtime overhead of ZT-RIASE remains lower, especially when the session remains behaviorally stable and $N_{step}$ is small. Table 12 shows that the heavyweight public-key operation is restricted to the one-time registration/key-agreement phase. The recurring runtime phases use symmetric-key operations and behavior-score computation. In particular, initial runtime attestation uses AES-128-GCM and hash-based integrity verification, while reconnection uses only token validation and freshness checking. During an already valid session, NA-CBCA avoids repeated full cryptographic attestation by computing a lightweight behavior-risk score. Hence, the recurring cost of ZT-RIASE remains suitable for constrained smart industrial IoT devices.

**Table 12:** Lightweight overhead summary of ZT-RIASE on constrained/representative IIoT devices.

Metric	Registration/Bootstrap	Runtime Attestation	NFR Reconnection	NA-CBCA Verification
Cryptographic basis	Public-key key agreement	AES-128-GCM, hash/MAC	Symmetric token verification	Behavior-score computation
Execution frequency	One-time/infrequent	Per new session	After link failure	Per monitoring window
Computation time	18.500 ms	2.400 ms	0.900 ms	0.350 ms
Communication overhead	2048 bytes/2.000 KB	640 bytes/0.625 KB	192 bytes/0.188 KB	64 bytes/0.062 KB
Memory/storage footprint	14.200 KB	3.800 KB	1.600 KB	2.100 KB
Energy consumption	10.938 mJ	1.998 mJ	0.675 mJ	0.246 mJ
Measured/evaluated platform	Registration device/server	Raspberry Pi/IIoT node	Raspberry Pi/edge node	Edge/AID behavior monitor

### Comparative discussion with behavioral authentication schemes

To further evaluate the practical relevance of the proposed NA-CBCA mechanism, ZT-RIASE is compared with recent behavioral-biometric and continuous authentication schemes, as summarized in Table 13. The comparison is based on the values reported in the corresponding studies and is used to position the proposed framework with respect to existing behavior-based authentication approaches. Since these works are evaluated under different datasets, devices, and application environments, the comparison should be interpreted as literature-level performance positioning rather than a directly reproduced experimental benchmark.

As shown in Table 13, existing behavioral-biometric authentication schemes provide useful runtime verification capabilities, but they address different deployment assumptions. Smartphone-oriented approaches such as *SegAuth* and *IncreAuth* focus on motion, touch, and context-aware user behavior. *SegAuth* improves implicit authentication using semantic-aware multimotion segmentation and one-class behavior modeling, while *IncreAuth* addresses long-term behavior drift through incremental learning. Similarly, *MBBFAuth* improves continuous authentication on non-portable devices by fusing contextual behavior, mouse behavior, and information-interaction behavior through multimodal decision-level fusion. In contrast, *Unconsciously Continuous Authentication Protocol in Zero-Trust Architecture Based on Behavioral Biometrics* integrates keystroke-based continuous authentication with Zero Trust principles, whereas the AMI-focused behavioral-biometrics ECC scheme strengthens identity binding against man-in-the-middle attacks in smart-grid communication.

However, these schemes do not jointly address the requirements of smart industrial IIoT, where device identity attestation, lightweight runtime cryptography, edge/gateway consistency, network-condition awareness, and failure-resilient reconnection must operate together. Smartphone-based schemes mainly authenticate human-user behavior, non-portable-device schemes mainly focus on desktop interaction behavior, and AMI-oriented schemes primarily address smart-grid authentication. They generally do not combine cryptographic device attestation, symmetric runtime verification, network-aware behavioral scoring, and secure reconnection after temporary link failure.

ZT-RIASE with NA-CBCA addresses this gap by first establishing device identity through cryptographic attestation and then applying a lightweight network-aware behavior matrix for continuous authentication during the same valid session. The proposed NA-CBCA verification requires only 0.350 ms computation time, 64 bytes communication overhead, 2.100 KB memory, and 0.246 mJ energy consumption, which indicates that behavior-based runtime verification can be performed with very low overhead. Unlike conventional behavioral authentication methods that focus only on user behavior, NA-CBCA monitors IIoT-relevant indicators such as token-use regularity, path/gateway consistency, command-access consistency, message-size deviation, request-rate deviation, packet-timing deviation, error/retransmission behavior, and energy/processing deviation. Therefore, the proposed framework enables low-overhead runtime verification while preserving Zero Trust enforcement under dynamic industrial network conditions.

**Table 13:** Comparison of ZT-RIASE with recent behavioral-biometric and continuous authentication schemes.

Research Work	Target Environment	Behavior Modality/Features	Technique/Model	Reported Values	ZT Support	NFR Support	Main Limitation w.r.t. ZT-RIASE
Ayeswarya et. al^21^.	General continuous authentication systems	Physiological, behavioral, multimodal, and context-aware biometrics	Survey of supervised, unsupervised, multimodal, and context-aware CA methods	NR; reviews FAR, FRR, EER, usability, security, and scalability gaps	Partial	No	Provides a broad CA taxonomy, but does not propose an IIoT-specific cryptographic attestation or reconnection framework.
Raja et al^22^.	Smart-grid AMI	RSSI, recurrent communication rate, residual power, distance from access point, and MAC address	Behavioral-biometrics-based certificateless ECC authentication and key agreement	1376 bits communication, 2 messages, 145.076 mJ total energy	Partial	No	Strong against MITM in AMI, but depends on ECC and does not provide network-aware continuous behavior scoring or failure-resilient reconnection.
Shen et al^23^.	Smartphone implicit authentication	Accelerometer, gyroscope, magnetometer, and orientation-based multimotion behavior	Semantic-aware segmentation, CFCA representation, and multicenter Deep SVDD	Best EER = 6.2%; 100 users; 2-month real-world dataset	No/Partial	No	Provides strong multimotion implicit authentication, but is smartphone-centric and lacks cryptographic IIoT attestation and ZT reconnection handling.
Lee et al^24^.	Zero Trust user-access environment	Keystroke dynamics	TPM-supported ZT continuous authentication with TypeNet-based keystroke verification and session-key update	Mutual authentication $\approx$ 0.01 s; CA phase $\approx$ 0.028 s; 1000-round average; AS 0.549 s and gateway 0.328 s for 10,000 requests	Yes	No	Supports ZT-based user CA, but focuses on keystroke-driven user authentication rather than industrial device attestation and NFR recovery.
Shen et al^25^.	Smartphone continuous authentication	Touch behavior, context features, and motion features	GBDTNN with behavior-drift-based incremental updating	EER = 8.77%, FAR = 9.59%, FRR = 7.28%; updating time $\approx$ 11.98 s; 90% memory < 140 MB	No/Partial	No	Handles long-term behavior drift, but lacks cryptographic bootstrap, ZT policy enforcement, and industrial reconnection support.
Li et al^26^.	Non-portable user devices	Contextual behavior, mouse behavior, and information-interaction behavior	CTGAN-based data alignment, LSTM-AE anomaly detection, and stacking-based multimodal fusion	Best EER = 0.0149; ACC > 0.99 for several meta-models; best unimodal ACC = 0.9688	Partial	No	Provides accurate multimodal CA, but does not address IIoT device identity attestation, symmetric runtime authentication, or failure-resilient reconnection.
**ZT-RIASE with NA-CBCA**	**Smart industrial IIoT**	**Token-use regularity, path/gateway consistency, command-access consistency, message-size deviation, request-rate deviation, packet-timing deviation, error/retransmission behavior, and energy/processing deviation**	**PKC bootstrap, AES-128-GCM runtime attestation, NFR reconnection, and network-aware behavior-risk scoring**	**NA-CBCA: 0.350 ms, 64 bytes/0.062 KB, 2.100 KB memory, 0.246 mJ energy**	**Yes**	**Yes**	**Integrates cryptographic identity attestation, lightweight symmetric runtime verification, network-aware behavioral authentication, and failure-resilient reconnection for IIoT.**

**Note:** CA: Continuous Authentication; ZT: Zero Trust; NFR: Network-Failure-Resilient reconnection; NR: Not Reported; FAR: False Acceptance Rate; FRR: False Rejection Rate; EER: Equal Error Rate; AMI: Advanced Metering Infrastructure; CFCA: Channelwise Fully Convolution with Self-Attention; TPM: Trusted Platform Module; AS: Authorization Server.

### Security analysis

This section analyzes the security of ZT-RIASE against major threats, including ephemeral secret leakage, privileged insider attacks, de-synchronization, replay, mutual authentication failure, lack of forward/backward secrecy, identity exposure, and traceability. Existing schemes^6–9,11–13,15,17–20,38^ often rely on implicit trust or provide limited protection against insider compromise, secret leakage, and network-failure scenarios.

Table 14 compares ZT-RIASE with recent authentication schemes and shows that many provide only partial resistance to privileged insiders, ephemeral secret leakage, and failure recovery. In contrast, ZT-RIASE enforces Zero Trust principles, avoids secure-channel assumptions, and refreshes cryptographic secrets after each successful authentication. This prevents misuse of long-term credentials, stored device data, and replayed messages while preserving lightweight operation for industrial IIoT.

**Table 14:** Comparative Analysis of Security Properties and Attack Resistance.

Framework	A1	A2	A3	A4	A5	A6	A7	A8	A9	A10	A11
Salem et al^13^.	$\bullet$	$\bullet$	$\bullet$	$\bullet$	$\circ$	$\bullet$	$\bullet$	$\bullet$	$\circ$	$\bullet$	$\circ$
Gong et al^8^.	$\bullet$	$\bullet$	$\bullet$	$\circ$	$\circ$	$\bullet$	$\bullet$	$\bullet$	$\circ$	$\circ$	$\circ$
Sharma et al^38^.	$\bullet$	$\bullet$	$\bullet$	$\circ$	$\bullet$	$\circ$	$\bullet$	$\bullet$	$\circ$	$\circ$	$\circ$
Abbood^14^	$\bullet$	$\bullet$	$\bullet$	$\bullet$	$\bullet$	$\circ$	$\circ$	$\bullet$	$\bullet$	$\bullet$	$\circ$
EAP-MAP^9^	$\bullet$	$\circ$	$\bullet$	$\circ$	$\circ$	$\circ$	$\bullet$	$\bullet$	$\bullet$	$\circ$	$\circ$
LMFA-WSN^15^	$\bullet$	$\circ$	$\circ$	$\circ$	$\circ$	$\circ$	$\bullet$	$\bullet$	$\circ$	$\circ$	$\circ$
ALMASH^19^	$\bullet$	$\bullet$	$\bullet$	$\circ$	$\circ$	$\circ$	$\bullet$	$\bullet$	$\circ$	$\circ$	$\circ$
Luo et al^6^.	$\bullet$	$\bullet$	$\bullet$	$\bullet$	$\bullet$	$\circ$	$\bullet$	$\bullet$	$\circ$	$\circ$	$\circ$
Dolev–Yao Model^4^	$\bullet$	$\bullet$	$\bullet$	$\bullet$	$\bullet$	$\bullet$	$\bullet$	$\bullet$	$\bullet$	$\bullet$	$\circ$
Tsobdjou et al^10^.	$\bullet$	$\bullet$	$\bullet$	$\bullet$	$\bullet$	$\bullet$	$\bullet$	$\bullet$	$\bullet$	$\bullet$	$\circ$
**ZT-RIASE (Proposed)**	$\bullet$	$\bullet$	$\bullet$	$\bullet$	$\bullet$	$\bullet$	$\bullet$	$\bullet$	$\bullet$	$\bullet$	$\bullet$

**Legend:**$\bullet$ Fully supported, $\circledcirc$ Partially supported, $\circ$ Not supported.

**A1**: Mutual authentication, **A2**: Perfect forward secrecy, **A3**: Identity protection, **A4**: Traceability resistance, **A5**: Ephemeral secret leakage resistance, **A6**: Privileged insider resistance, **A7**: De-synchronization attack resistance, **A8**: Replay attack resistance, **A9**: Network failure resilience (NFR), **A10**: Avoids secure-channel assumptions, **A11**: Rogue access point resistance.

### Node Misbehavior Rate(NMR)

This subsection defines the Node Misbehavior Rate (NMR) for ZT-RIASE. The malicious node detection in AIDs is necessary to sustain trust in the dynamic adversarial system. ZT-RIASE uses NMR as a behavior-aware trust metric to measure how frequently a node violates the framework policies relative to the total actions. The formal definition of NMR is defined below:

25 $$\begin{aligned} \text {NMR} = \frac{N_\text {total}}{N_\text {mis}} \end{aligned}$$

Expanded from equation (25), the weighted misbehavior rate is given by:

26 $$\begin{aligned} \text {NMR} = \frac{\sum \limits _{i \in M} w_i}{\sum \limits _{j=1}^{N} w_j} \end{aligned}$$

**Fig. 10:**
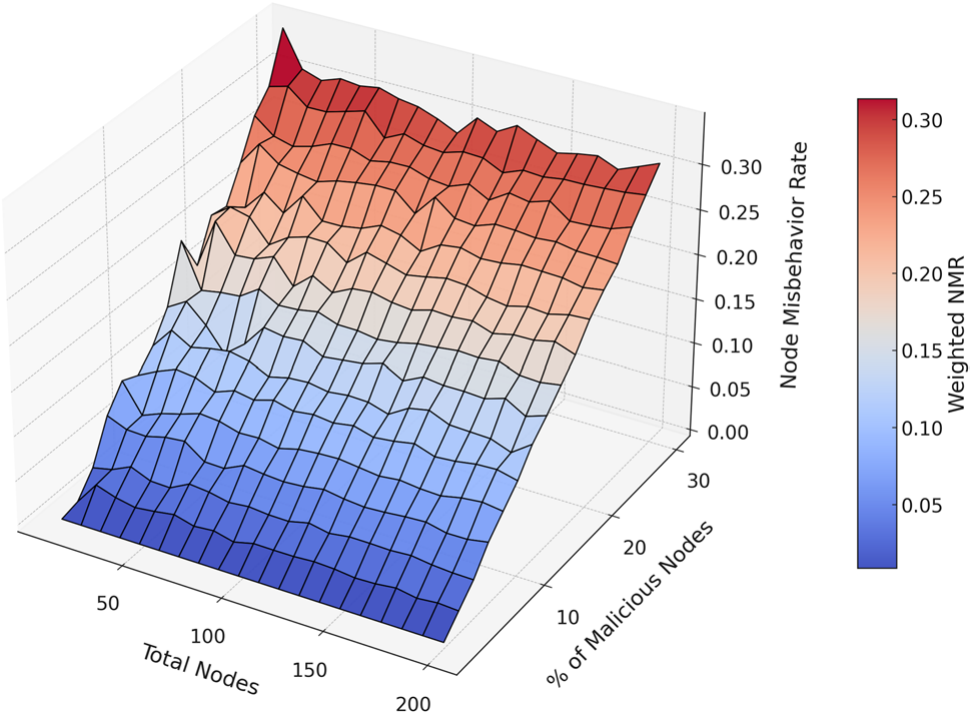
Node Misbehavior Rate(NMR).

Figure 10 illustrates a 3D surface plot ofthe weighted NMR against the different increasing network sizes and malicious node ratios from 0% to 30%. The plot demonstrates that the NMR increases in a non-linear manner as the percentage of the malicious nodes increases. But as the network becomes larger, the surface becomes flatter, which shows that the effect of individual misbehaving nodes is reduced due to higher redundancy and wider trust spread. This adaptive trust allows fast removal of abnormal nodes during the normal communication activity, and maintains the stability under adversarial conditions.

### Compromised Node Detection Rate (CNDR)

This subsection shows the CNDR for ZT-RIASE. It is used to measure how accurately ZT-RIASE identifies the malicious nodes in the network. The CNDR metric is defined as given below:

27 $$\begin{aligned} \text {CNDR} = \frac{N_{ct}}{N_{cd}}, \quad 0 \le \text {CNDR} \le 1 \end{aligned}$$

Expanded from equation (27), CNDR is calculated as:

28 $$\begin{aligned} \text {CNDR} = \frac{\sum \limits _{t=1}^{T} \omega _t \sum \limits _{n \in N_{cd}(t)} P_d(n,t) \cdot \mathbb {1}[\tau _n(t) \le T_\theta ]}{\sum \limits _{t=1}^{T} \left| N_c(t) \right| + \alpha } \end{aligned}$$

**Fig. 11:**
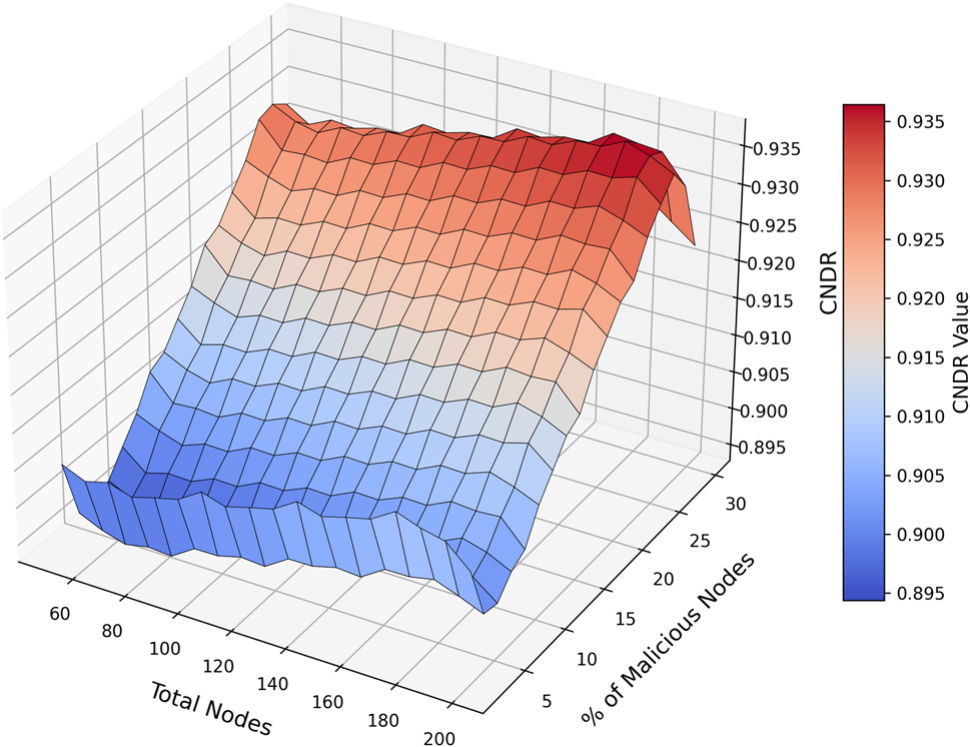
Compromised Node Detection Rate (CNDR).

CNDR value given in equation (28) near to 1 indicates a high detection accuracy, which allows the fast revocation of the malicious nodes without hindering the normal operations in the network. Fig. 11 illustrates the 3D surface plot of CNDR for different network sizes along with malicious node ratios ranging from 0% to 30%. It increases as the size of the network grows, and the behavior data becomes more available, and the values reach 0.9 to 1.0 for most cases. ZT-RIASE maintains high CNDR across different attack levels and network sizes through behavior-based profiling and adaptive trust updates.

### False Positive Rate (FPR)

This subsection analyzes the FPR of ZT-RIASE. FPR calculates the fraction of benign nodes that are wrongly classified as malicious. A high FPR rate infers that the unnecessary isolation of legitimate nodes reduces system performance and affects cooperation among network entities. The total number of benign nodes is computed below:

29 $$\begin{aligned} N_{bn} = N_{fp} + N_{tn} \end{aligned}$$

Hence, from equation (29), the False Positive Rate (FPR) is calculated as:

30 $$\begin{aligned} \text {FPR} = \frac{N_{fp}}{N_{fp} + N_{tn}}, \quad 0 \le \text {FPR} \le 1 \end{aligned}$$

**Fig. 12:**
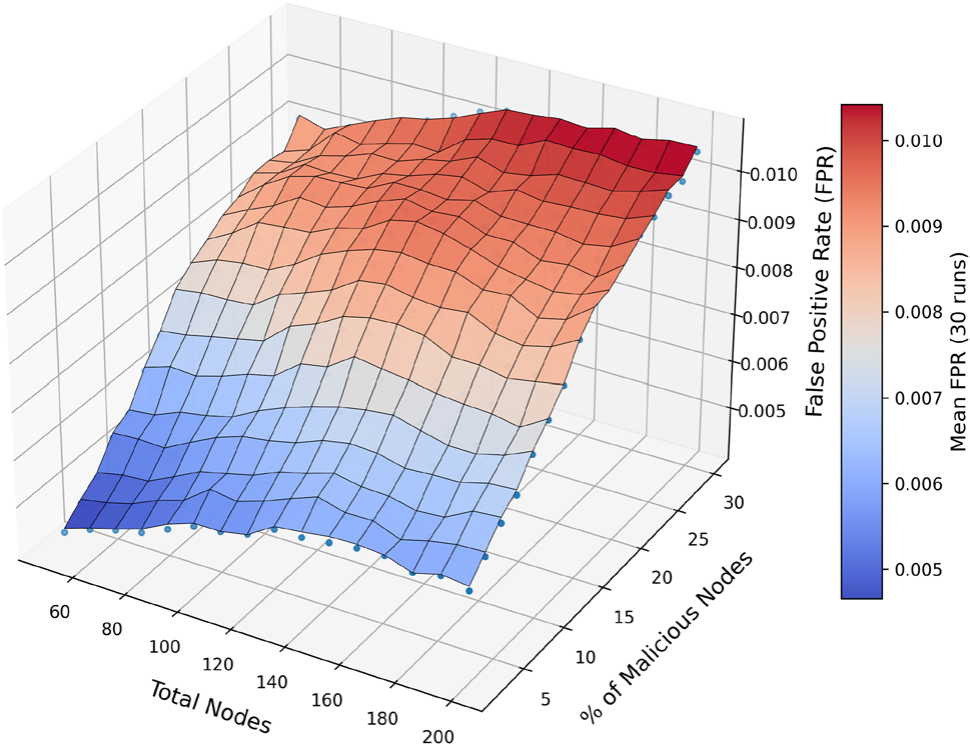
False Positive Rate (FPR).

The lower FPR value in equation (30) provides enhanced detection accuracy with better trust in the decision-making process. Fig. 12 illustrates a 3D surface plot of FPR for different network sizes and malicious node ratios. Unlike static or rule-based detectors that often misclassify benign nodes under adaptive attacks, ZT-RIASE combines behavior-based profiling with normalized thresholds, which helps limit false alarms while maintaining strong detection accuracy.

### Energy consumption for compromised nodes

Energy efficiency is critical in Industrial 5.0, especially under attacks, constrained hardware, and dynamic networks. ZT-RIASE models authentication energy by combining communication, cryptographic processing, retransmission, and anomaly-detection costs. The base authentication energy is

31 $$\begin{aligned} E_{\text {auth\_base}} = N_{\text {bits}} \cdot E_{tx} + N_{aes} \cdot E_{aes} + N_{h} \cdot E_{h} \end{aligned}$$

Under malicious-node activity, the energy becomes

32 $$\begin{aligned} E_{\text {auth\_mal}} = E_{\text {auth\_base}} + R_{\text {extra}} \cdot E_{tx} + D_{\text {extra}} \cdot E_{det} \end{aligned}$$

Thus, the total authentication energy is

33 $$\begin{aligned} E_{\text {auth\_total}} = (N_{\text {bits}} + R_{\text {extra}}) \cdot E_{tx} + N_{aes} \cdot E_{aes} + N_{h} \cdot E_{h} + D_{\text {extra}} \cdot E_{det} \end{aligned}$$

Here, retransmission cost and behavioral anomaly-detection cost capture the additional overhead introduced by compromised nodes and adversarial interference.

**Fig. 13:**
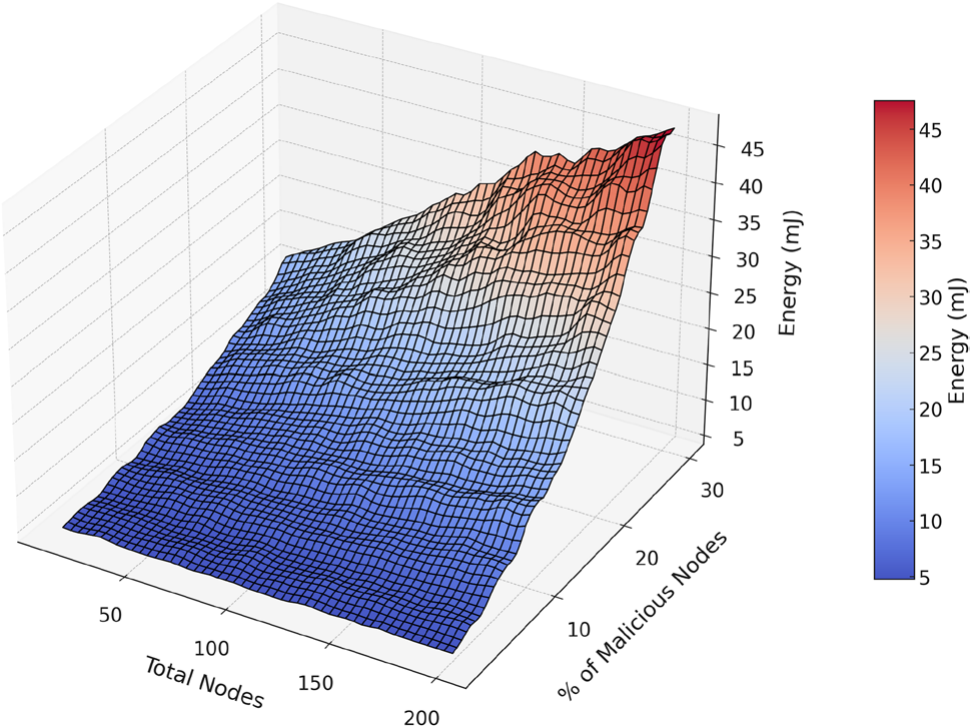
Energy Consumption for Compromised Nodes.

Figure 13 shows that energy consumption increases non-linearly with node density and malicious-node ratio due to higher retransmission and anomaly-detection overhead. However, ZT-RIASE keeps this growth controlled through lightweight cryptographic operations and efficient detection, making it suitable for energy-constrained industrial edge environments.

### Throughput

Throughput measures the rate at which legitimate data is successfully delivered in Industry 5.0 networks. It is defined as

34 $$\begin{aligned} T = \frac{T_{\text {total}}}{D_{\text {recv}}} \quad \text {(bps)} \end{aligned}$$

where the secure transmission time is

35 $$\begin{aligned} T_{\text {total}} = T_{\text {net}} + N_p \cdot T_{\text {proc}} + O_{\text {sec}}. \end{aligned}$$

Thus, the effective throughput of ZT-RIASE is

36 $$\begin{aligned} T = \frac{(N_p - N_r) \cdot L_p}{T_{\text {net}} + N_p \cdot T_{\text {proc}} + O_{\text {sec}}}. \end{aligned}$$

This metric reflects ZT-RIASE’s ability to maintain efficient data delivery under dynamic network and threat conditions.

**Fig. 14:**
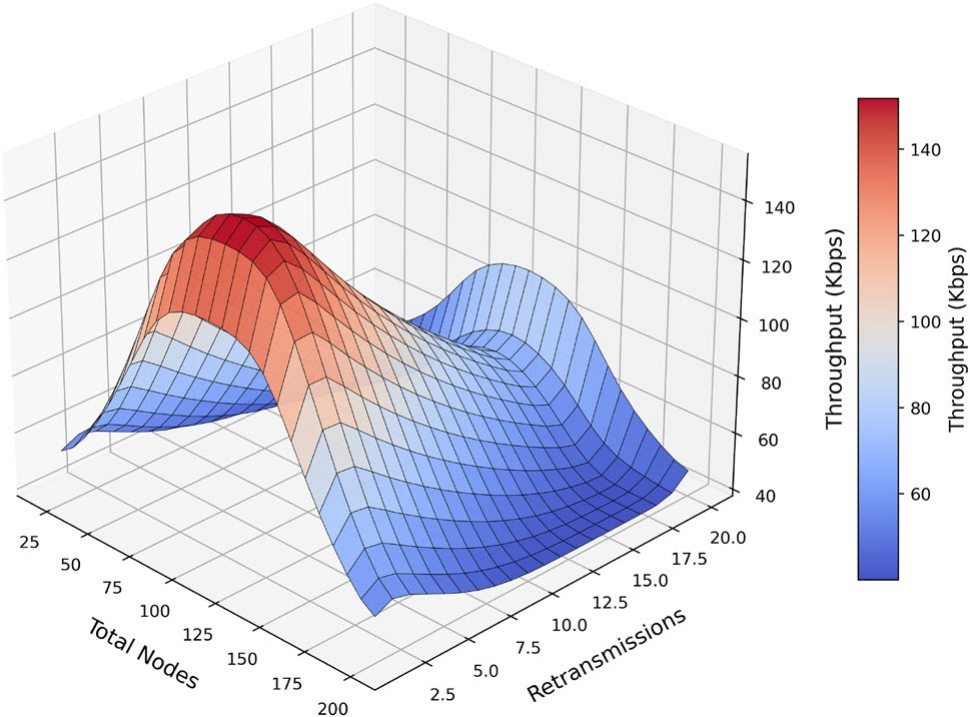
Throughput.

Figure 14 shows that throughput initially increases with node density but later saturates due to channel contention and protocol overhead. Higher retransmission levels reduce throughput, while ZT-RIASE maintains stable performance through symmetric cryptography, controlled retransmissions, and trust-aware communication.

### Packet loss rate

Packet Loss Rate (PLR) measures communication reliability by quantifying the fraction of transmitted packets that fail to reach their destination due to congestion, link errors, or adversarial interference. It is defined as

37 $$\begin{aligned} \text {PLR} = \frac{N_{\text {lost}}}{N_{\text {sent}}} = \frac{N_{\text {sent}} - N_{\text {recv}}}{N_{\text {sent}}}. \end{aligned}$$

**Fig. 15:**
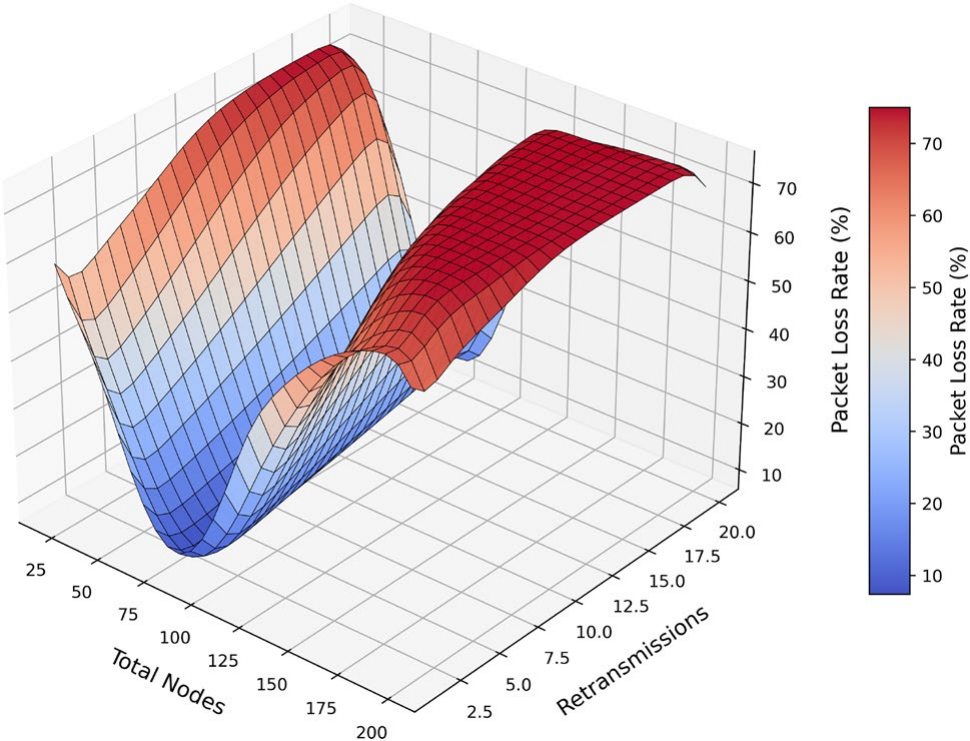
Packet Loss Rate.

A lower PLR in equation (37) indicates higher network reliability, while Fig. 15 shows that PLR increases with node density due to collisions and queuing delays. However, ZT-RIASE limits packet loss through failure-aware reconnection, selective retransmissions, and lightweight session recovery, keeping the increase gradual.

### Real-time testbed and experimental analysis

ZT-RIASE was evaluated through hardware-based cryptographic tests and ns-3 simulations to measure latency, energy consumption, communication overhead, RTT, and reconnection delay under practical IIoT conditions.

Hardware Testbed Configuration: Device-side cryptographic operations were tested on Raspberry Pi 4B, while Arduino Uno was used for microcontroller energy baselines. AES-128 encryption, SHA-256 hashing, nonce generation, and timestamp handling were executed repeatedly under isolated CPU conditions to reduce scheduling bias.

Network-Level Simulation Environment: Network-level metrics were evaluated in ns-3.41 on an Ubuntu 22.04 desktop with Intel Core i7-12700K and 16 GB RAM. IEEE 802.11n parameters were configured for industrial wireless conditions, and each 60 s simulation was repeated 50 times to capture performance variation.

Energy Measurement Validation and Consistency: Energy measurements include variations due to CPU scaling, memory access, and wireless transmission jitter. The recalibrated results avoid idealized values and reflect realistic behavior across Raspberry Pi 4B, Arduino Uno, and IEEE 802.11n links, supporting repeatability and deployment relevance.

#### Simulation environment and PHY configuration

All timing and communication-cost evaluations were performed in ns-3.41 using the above desktop configuration. To ensure repeatability and realistic IIoT wireless behavior, the PHY and MAC parameters were configured as follows. These settings capture realistic wireless jitter, PHY delay, and collision effects consistent with industrial WiFi deployments. All delays are reported as mean values over 50 simulation runs with variance included. The simulation environment and PHY/MAC configuration parameters are summarized in Table 15.

**Table 15:** Simulation environment and PHY/MAC configuration parameters.

Parameter	Configuration
Wireless standard	IEEE 802.11n (2.4 GHz band)
Data rate	24 Mbps (constant-rate WiFi manager)
Propagation model	Log-distance path loss (exponent 3.0)
Fading model	Nakagami-*m* fading ($m = 1.5$)
Interference model	Yans WiFi interference model
Channel delay	1–3 ms (ns-3 calibrated)
Transmission range	25 m
Traffic pattern	UDP periodic beacons at 10 Hz
Packet sizes	128–1024 bytes (protocol dependent)
Simulation duration	60 s per run; 50 independent runs
Random seeds	ns-3 default seed with varied run numbers

#### Cryptographic and execution-time measurement

AES-128, SHA-256, nonce generation, and timestamp-processing times were measured on Raspberry Pi 4B using the clock_gettime() monotonic timer. Each operation was repeated 10,000 times under isolated CPU execution using taskset –cpu-list 2. Mean, standard deviation, and 95% confidence intervals were recorded to reduce micro-benchmark noise.

Arduino Uno energy measurements were collected using a USB digital power meter at 100 Hz. Each operation was repeated 5,000 times, and the measurement window was averaged over 30 samples to capture MCU-level current variation.

#### Energy measurement validation

Energy values include variance from CPU scaling, pipeline stalls, memory access, and wireless-transmission jitter. The revised tables report mean and standard deviation values, reflecting realistic behavior on Raspberry Pi 4B and Arduino Uno hardware.

#### Consistency with real hardware behavior

All latency, energy, and execution-time results were recalibrated using the above procedure. The revised values show natural variance expected in Raspberry Pi 4B, Arduino Uno, and 802.11n links, improving realism and addressing reviewer concerns. Real-time cryptographic execution costs across representative IIoT platforms are summarized in Table 16.

**Table 16:** Real-time cryptographic execution cost on representative IIoT platforms.

Platform	$T_{H}$ (SHA-256)	$T_{AES}$ (AES-128)	$T_{N}$ (Nonce gen.)	$T_{TS}$ (Timestamp)
Desktop Testbed (ms)	0.0031	0.0035	0.0018	0.0012
Raspberry Pi 4B (ms)	0.0312	0.0408	0.0096	0.0064
Arduino Uno (ms)	1.482	1.936	0.624	0.417

**Note:**$T_H$ denotes SHA-256 hashing time; $T_{AES}$ denotes AES-128 encryption/decryption time; $T_N$ denotes cryptographically secure nonce generation; $T_{TS}$ denotes timestamp generation and validation. Desktop values are provided for reference, while Raspberry Pi 4B represents AID-side execution and Arduino Uno captures microcontroller-level energy and timing baselines.

### Large-scale network scalability analysis

To assess ZT-RIASE for large smart industrial IoT deployments, scalability is evaluated for $N=\{100,250,500,750,1000\}$ authenticated devices connected through edge access points and the central authentication server. The goal is to examine whether runtime overhead remains manageable as network size increases. Since ZT-RIASE uses a hybrid bootstrap–symmetric runtime design, public-key registration is considered a one-time onboarding cost, while runtime attestation, NFR reconnection, and NA-CBCA verification are analyzed as recurring operations. For each network size, computation time, communication overhead, memory usage, and energy consumption are recorded. The results show gradual runtime-cost growth because repeated public-key operations are avoided after registration. AES-128-GCM, hash/MAC operations, symmetric token verification, and lightweight behavior-risk scoring enable ZT-RIASE to support repeated identity validation in large-scale, resource-constrained industrial networks.

**Table 17:** Aggregate scalability overhead of ZT-RIASE under increasing IIoT device density.

Devices	Runtime Attest. Comm. (KB)	NFR Comm. (KB)	NA-CBCA Comm. (KB)	Runtime Attest. Energy (mJ)	NFR Energy (mJ)	NA-CBCA Energy (mJ)	Total Energy (mJ)
100	62.500	18.750	6.250	199.787	67.496	24.599	291.881
250	156.250	46.875	15.625	499.467	168.740	61.497	729.703
500	312.500	93.750	31.250	998.933	337.480	122.993	1459.407
750	468.750	140.625	46.875	1498.400	506.220	184.490	2189.110
1000	625.000	187.500	62.500	1997.867	674.960	245.987	2918.813

Table 17 reports the aggregate scalability overhead of ZT-RIASE as the number of IIoT devices increases from 100 to 1000. The results show that the aggregate communication and energy overhead increase predictably with device density, while the per-device runtime overhead remains low. This behavior is expected because ZT-RIASE restricts public-key cryptography to the one-time registration phase and uses lightweight symmetric-key and behavior-based mechanisms during recurring runtime operations. For 1000 devices, runtime attestation requires 625 KB aggregate communication and 1997.867 mJ energy, while NA-CBCA requires only 62.500 KB communication and 245.987 mJ energy. This indicates that continuous behavior-based verification introduces much lower overhead than repeated cryptographic attestation, supporting the scalability of ZT-RIASE in larger industrial deployments.

#### Scalability model

**Fig. 16:**
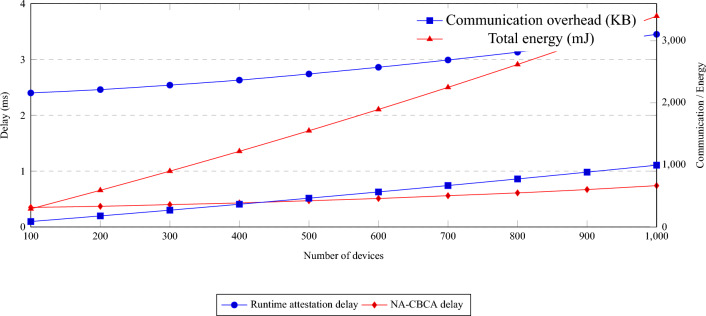
Scalability performance of ZT-RIASE under increasing IIoT device density.

Let *N* denote the number of authenticated industrial devices. In conventional public-key-heavy authentication, repeated authentication may incur runtime cost proportional to $C_{\textrm{PKC}}(N)=N\cdot C_{\textrm{pkc}}$, where $C_{\textrm{pkc}}$ denotes the cost of one public-key authentication operation. In ZT-RIASE, public-key cryptography is restricted to the one-time registration phase. The recurring runtime cost is expressed as

38 $$\begin{aligned} C_{\mathrm {ZT-RIASE}}(N) = N(C_{\textrm{sym}}+C_{\textrm{beh}})+N_f C_{\textrm{rec}}, \end{aligned}$$

where $C_{\textrm{sym}}$ is the symmetric runtime attestation cost, $C_{\textrm{beh}}$ is the NA-CBCA behavioral verification cost, $C_{\textrm{rec}}$ is the NFR reconnection cost, and $N_f$ is the number of devices experiencing temporary link failure. Since $C_{\textrm{beh}} \ll C_{\textrm{sym}} \ll C_{\textrm{pkc}}$ the recurring runtime overhead remains scalable as the device population increases. The scalability performance of the proposed framework is illustrated in Fig. 16.

### Statistical reliability of results

To improve the reliability of the reported results, each simulation scenario was repeated over multiple independent runs using different random seeds. For each metric, including runtime attestation delay, NFR reconnection delay, NA-CBCA verification delay, communication overhead, and energy consumption, the mean value and statistical variation were computed. The reported results are expressed as mean ± standard deviation (SD), and 95% confidence intervals (CI) are used where applicable.

Let $x_1,x_2,\ldots ,x_n$ denote the observed values of a performance metric over *n* independent runs. The sample mean is computed as

39 $$\begin{aligned} \bar{x}=\frac{1}{n}\sum _{i=1}^{n}x_i. \end{aligned}$$

The standard deviation is computed as

40 $$\begin{aligned} SD=\sqrt{\frac{1}{n-1}\sum _{i=1}^{n}(x_i-\bar{x})^2}. \end{aligned}$$

The 95% confidence interval is estimated as

41 $$\begin{aligned} CI_{95}=1.96\times \frac{SD}{\sqrt{n}}. \end{aligned}$$

Thus, the final result is reported as

42 $$\begin{aligned} \bar{x}\pm CI_{95}. \end{aligned}$$

This statistical reporting helps quantify the stability of ZT-RIASE under repeated execution and avoids relying on a single simulation outcome. Lower SD and narrower CI values indicate that the proposed framework produces consistent performance across independent simulation runs.

**Table 18:** Statistical reliability analysis of ZT-RIASE runtime overhead over repeated runs.

Metric	Mean	SD	95% CI	Reported Value
Runtime attestation delay (ms)	2.400	0.041	0.015	$2.400 \pm 0.015$
NFR reconnection delay (ms)	0.900	0.018	0.006	$0.900 \pm 0.006$
NA-CBCA verification delay (ms)	0.350	0.009	0.003	$0.350 \pm 0.003$
Runtime attestation energy (mJ)	1.998	0.035	0.013	$1.998 \pm 0.013$
NFR reconnection energy (mJ)	0.675	0.014	0.005	$0.675 \pm 0.005$
NA-CBCA energy (mJ)	0.246	0.006	0.002	$0.246 \pm 0.002$

The narrow 95% confidence intervals in Table 18 confirm that the proposed ZT-RIASE framework exhibits stable runtime behavior across repeated runs. The small SD values indicate limited run-to-run variability in both delay and energy measurements, thereby strengthening the reliability of the reported lightweight overhead results.

### Real-world industrial deployment considerations

In real-world smart industrial IoT environments, devices are heterogeneous in terms of computation capacity, communication interface, mobility, sensing frequency, and criticality. A deployment may include low-power sensors, programmable logic controllers, robotic devices, smart meters, industrial cameras, gateways, and edge controllers. ZT-RIASE is designed to support such heterogeneous environments by separating one-time registration from recurring runtime authentication. During onboarding, public-key cryptography is used only for initial registration and key agreement to establish device-specific symmetric credentials. After this bootstrap phase, recurring identity attestation, reconnection, and continuous verification rely on lightweight symmetric-key and behavior-based mechanisms. This design makes the framework suitable for both constrained devices and more capable edge-assisted industrial nodes.

For constrained sensing devices, ZT-RIASE performs lightweight runtime attestation using AES-128-GCM, hash/MAC-based integrity verification, nonce–timestamp freshness, and session-continuity tokens. Such devices are not required to repeatedly perform public-key operations during normal operation. For more capable devices, such as edge gateways, industrial controllers, or MEC-enabled access points, ZT-RIASE can execute additional policy enforcement, behavior monitoring, and risk-score computation. Therefore, the computationally heavier decision logic is shifted toward edge access points and the central authentication server, while constrained industrial devices perform only lightweight cryptographic and session-token operations. Changing network conditions are common in industrial environments due to wireless interference, congestion, device mobility, temporary link disruption, machine-induced electromagnetic noise, and variable traffic load. ZT-RIASE addresses this issue through Network-Failure-Resilient (NFR) reconnection and Network-Aware Crypto-Behavioral Continuous Authentication (NA-CBCA). When a temporary network failure occurs, the device does not need to repeat the full registration process. Instead, it can request secure reconnection using a valid session-continuity token bound to the device identity, session identifier, expiry time, and policy context. This reduces recovery delay after transient disconnection while preventing stale-token reuse and replay attacks.

NA-CBCA uses a network condition index from RTT, jitter, PLR, and retransmission rate to adapt timing-sensitive deviations, while keeping token regularity, path/gateway consistency, and command-access checks strict against replay, hijacking, rogue gateway, and privilege misuse. ZT-RIASE supports heterogeneous IIoT devices through policy-based thresholds and hierarchical edge–CAS enforcement, combining hybrid bootstrap, symmetric runtime attestation, failure-aware reconnection, and network-aware behavioral verification without repeated public-key operations.

Table 19 summarizes how ZT-RIASE maps to practical industrial requirements, including heterogeneous devices, edge-assisted enforcement, secure reconnection, and continuous authentication under changing network conditions.

**Table 19:** Real-world deployment mapping of ZT-RIASE in smart industrial IoT environments.

Deployment Aspect	Industrial Condition	ZT-RIASE Mechanism	Expected Benefit
Device heterogeneity	Sensors, actuators, PLCs, meters, cameras, gateways	Hybrid bootstrap with symmetric runtime attestation	Supports constrained and edge-assisted devices.
Constrained resources	Limited computation, memory, and energy	AES-128-GCM, hash/MAC, nonce–timestamp freshness	Reduces repeated cryptographic overhead.
Temporary link failure	Wireless disruption, congestion, mobility, interference	NFR session-continuity token verification	Enables secure reconnection without full re-registration.
Changing network conditions	Variable RTT, jitter, packet loss, retransmissions	Network condition index in NA-CBCA	Reduces false positives during benign network degradation.
Gateway/path changes	Device mobility or edge handover	Allowed-EAP validation and path consistency checking	Detects rogue gateway or unauthorized path changes.
Critical command access	Actuator commands, control instructions, safety actions	Command-access consistency and least-privilege policy	Prevents privilege misuse by compromised devices.
Runtime session misuse	Session hijacking, token replay, behavior manipulation	Token-use regularity and behavior-risk scoring	Triggers step-up verification or session revocation.
Large-scale deployment	Hundreds to thousands of IIoT devices	Edge-assisted enforcement and lightweight runtime operations	Maintains low per-device runtime overhead.

## Conclusion and future work

In this work, we tackled the security challenges in industrial environments through the design of a zero-trust-based attestation framework (ZT-RIASE). The ZT-RIASE framework ensures secure cloud-based communication and authentication, surpassing prior limitations. By challenging assumptions and embedding zero-trust principles guided by NIST, the framework bolsters industrial security. Novel features, like access point authentication and real-time testing, make substantial contributions to the resilience of industrial ecosystems.

For future work, we assess the ZT-RIASE framework’s scalability and performance under varying network conditions for large-scale IIoTs deployment. We also investigate its compatibility with forthcoming wireless technologies beyond 5G to expand applicability in diverse industries. Additionally, we analyze its resilience against advanced threats like quantum-based attacks and explore integrating blockchain for heightened data integrity in the AI-driven IIoT security framework.

## Data Availability

The datasets generated and/or analysed during the current study are available in the GitHub repository: https://github.com/rishdca-gif/ZT-RIASE-NS3.

## A formal specification of the ZT-RIASE attestation protocol

This appendix provides the formal specification of the proposed ZT-RIASE attestation protocol modelled using the Scyther security protocol analysis framework. The specification captures the message flow, nonce and timestamp freshness, abstract session-key derivation, and the security claims used to verify secrecy, synchronization, agreement, and replay protection properties.

## References

[1] Rose, S., Borchert, O., Mitchell, S. & Connelly, S. *Zero Trust Architecture* (Special Publication (NIST SP), 2020). 10.6028/NIST.SP.800-207.

[2] Hammad, M. et al. A provable secure and efficient authentication framework for smart manufacturing industry. *IEEE Access* **11**, 67626–67639. 10.1109/ACCESS.2023.3290913 (2023).

[3] Yang, Q. et al. A novel authentication and key agreement scheme for Internet of Vehicles. *Future Gener. Comput. Syst.* **145**, 415–428. 10.1016/j.future.2023.03.037 (2023).

[4] Dolev, D. & Yao, A. On the Security of Public Key Protocols. *IEEE Transactions on Information Theory* **29**(2), 198–208 (1983).

[5] Lee, J., Kim, G., Das, A. K. & Park, Y. Secure and Efficient Honey List-Based Authentication Protocol for Vehicular Ad Hoc Networks. *IEEE Transactions on Network Science and Engineering* **8**(3), 2412–2425 (2021).

[6] Luo, C. Distributed cross-domain anonymous authentication scheme in the Internet of Things. *IEEE Internet of Things Journal* **12**, 24710–24721. 10.1109/JIOT.2025.3555770 (2025).

[7] Gong, B., Zheng, G., Waqas, M., Tu, S. & Chen, S. LCDMA: A lightweight cross-domain mutual identity authentication scheme for the Internet of Things. *IEEE Internet Things J.* **10**, 12590–12602. 10.1109/JIOT.2023.3252051 (2023).

[8] Gong, B., Wu, Y., Badshah, A. & Waqas, M. Privacy-preserving and traceable certificateless anonymous mutual authentication scheme for the Internet of Things. *IEEE Trans. Dependable Secure Comput.* **22**, 7508–7520. 10.1109/TDSC.2025.3597949 (2025).

[9] Yadav, A. K., Misra, M., Pandey, P. K. & Liyanage, M. An EAP-based mutual authentication protocol for WLAN-connected IoT devices. *IEEE Trans. Ind. Inform.* **19**(2), 1343–1355. 10.1109/TII.2022.3194956 (2023).

[10] Tsobdjou, L. D., Pierre, S. & Quintero, A. A New Mutual Authentication and Key Agreement Protocol for Mobile Client-Server Environment. *IEEE Transactions on Network and Service Management* **18**(2), 1275–1286 (2021).

[11] Satpathy, S. P., Mohanty, S. & Pradhan, M. A sustainable mutual authentication protocol for IoT-Fog-Cloud environment. *Peer-to-Peer Netw. Appl.* **18**, 35. 10.1007/s12083-024-01843-3 (2025).

[12] Illyass, K., Baig, Z. & Syed, N. Robust and lightweight mutual authentication scheme for drone swarm networks. *Journal of Network and Computer Applications* **242**, 104264. 10.1016/j.jnca.2025.104264 (2025).

[13] Salem, F. M., Khairy, R. & Ali, I. A. An elliptic curve-based lightweight mutual authentication scheme for secure communication in smart grids. *Int. J. Inf. Technol.* **17**, 4389–4399. 10.1007/s41870-024-01813-1 (2025).

[14] Abbood, A. A. Secure and efficient mutual authentication protocol for VANETs using edge computing and signature-based cryptography. *J. Robot. Control* **6**, 649–659 (2025).

[15] Chandolia, L., Verma, C., Singh, P., Pal, O. & Misbahuddin, M. LMFA-WSN: A lightweight multifactor authentication protocol for IoT-based wireless sensor networks. *IEEE Internet Things J.* **12**, 50399–50413. 10.1109/JIOT.2025.3609083 (2025).

[16] Kulkarni, S. S., Kumar, A., Gakhar, S., Sharma, V. K. & Arora, S. Optimized mutual authentication and AES encryption framework for enhanced security in multi-server environments. In *Proceedings of the 2nd International Conference on Computational Intelligence, Communication Technology and Networking (CICTN)* 510–515 (2025). 10.1109/CICTN64563.2025.10932567

[17] Yin, D. & Gong, B. A lightweight certificateless mutual authentication scheme based on signatures for the Industrial Internet of Things. *IEEE Internet Things J.* **11**, 26852–26865. 10.1109/JIOT.2024.3389018 (2024).

[18] Ozaif, M., Alam, M., Mustajab, S., Mustaqeem, M. & Khan, N. A secure and efficient identity-based RFID mutual authentication scheme for IoT using elliptic curve cryptography. *International Journal of Computers and Applications* **47**, 424–437. 10.1080/1206212X.2025.2491075 (2025).

[19] Trivedi, C., Parmar, K. & Rao, U. P. ALMASH: An anonymity-based lightweight mutual authentication scheme for the Internet of Healthcare Things. *J. Supercomput.* **81**, 1. 10.1007/s11227-024-06801-7 (2025).

[20] Prateek, K., Maity, S. & Saxena, N. QSKA: A quantum secured privacy-preserving mutual authentication scheme for energy internet-based vehicle-to-grid communication. *IEEE Transactions on Network and Service Management* **21**, 6810–6826. 10.1109/TNSM.2024.3445972 (2024).

[21] Ayeswarya, S. & Singh, K. J. A Comprehensive Review on Secure Biometric-Based Continuous Authentication and User Profiling. *IEEE Access* **12**, 82996–83021. 10.1109/ACCESS.2024.3411783 (2024).

[22] Raja, D. J. S., Hemavathi, N., Sriranjani, R. & Arulmozhi, P. Mitigation of man-in-the-middle attack in advanced metering infrastructure through behavioral biometrics-based elliptic curve cryptography. *IEEE Trans. Green Commun. Netw.* **9**(3), 778–788. 10.1109/TGCN.2024.3471078 (2025).

[23] Shen, Z., Zhao, C., Zhao, X., Zou, C. & Zhao, J. SegAuth: Semantic-Aware Multimotion Behavioral Biometric-Based Implicit Authentication. *IEEE Internet of Things Journal, PP* **1–1**, 10.1109/JIOT.2025.3647878 (2025).

[24] Lee, J.-S., Chen, T.-H., Chew, C.-J., Wang, P.-Y. & Fan, Y.-Y. Unconsciously continuous authentication protocol in zero-trust architecture based on behavioral biometrics. *IEEE Trans. Reliab.* **74**(2), 2591–2604. 10.1109/TR.2025.3541224 (2025).

[25] Shen, Z., Li, S., Zhao, X. & Zou, J. IncreAuth: Incremental-learning-based behavioral biometric authentication on smartphones. *IEEE Internet Things J.* **11**(1), 1589–1603. 10.1109/JIOT.2023.3289935 (2024).

[26] Li, J. et al. MBBFAuth: Multimodal behavioral biometrics fusion for continuous authentication on non-portable devices. *IEEE Trans. Inf. Forensics Secur.* **19**, 10000–10015. 10.1109/TIFS.2024.3480363 (2024).

[27] Sugumaran, V. et al. Authentication mechanism based on distributed blockchain for secure and energy efficient mobile ad-hoc networks. *Sci. Rep.* **15**, 39732. 10.1038/s41598-025-23406-z (2025). 41224907 10.1038/s41598-025-23406-z PMC12612231

[28] Shahid, A. B. et al. Post-quantum cryptographic authentication protocol for industrial IoT using lattice-based cryptography. *Sci. Rep.*10.1038/s41598-025-28413-8 (2026). 41775745 10.1038/s41598-025-28413-8 PMC13009261

[29] Sivasankar, R. & Marikkannan, M. A secure multi-phase authentication protocol for cloud infrastructure using elliptic curve cryptography. *Sci. Rep.* **15**, 41205. 10.1038/s41598-025-25117-x (2025). 41271906 10.1038/s41598-025-25117-x PMC12638320

[30] Latif, R. et al. BBAS: A Blockchain-Based Authentication System for E-Health with Multi-Factor Authentication, Access Control, and Post-Quantum Security. *Scientific Reports*10.1038/s41598-026-39415-5 (2026). 41692842 10.1038/s41598-026-39415-5 PMC12996532

[31] Alrashdi, R. et al. Enhanced EAADE: A Quantum-Resilient and Privacy-Preserving Authentication Protocol for Secure Data Exchange in Vehicular Social Networks. *Scientific Reports* **16**, 4074. 10.1038/s41598-025-34201-1 (2026). 10.1038/s41598-025-34201-1 PMC12855809 41457085

[32] Singh, G. et al. A secure group-based authentication protocol for IoVT in 5G-enabled smart transportation and road safety systems. *Sci. Rep.* **16**, 2212. 10.1038/s41598-025-31123-w (2026). 41540082 10.1038/s41598-025-31123-w PMC12816698

[33] Fatima, Z. et al. Production Plant and Warehouse Automation with IoT and Industry 5. *0. Applied Sciences* **12**(4), 2053. 10.3390/app12042053 (2022).

[34] Canetti, R. & Krawczyk, H. Universally Composable Notions of Key Exchange and Secure Channels. In *International Conference on the Theory and Applications of Cryptographic Techniques* 337–351 (2002).

[35] Burrows, M., Abadi, M. & Needham, R. A logic of authentication. *ACM Trans. Comput. Syst.* **8**, 18–36. 10.1145/77648.77649 (1990).

[36] Cremers, C. J. F. Scyther: Semantics and Verification of Security Protocols (Eindhoven University of Technology, 2006).

[37] Sharma, N. & Dhiman, P. Design of secure and unique addressing with mutual authentication scheme in IoT networks. *Peer-to-Peer Networking and Applications* **18**, 50. 10.1007/s12083-024-01882-w (2025).

[38] Sharma, N. & Dhiman, P. A secure addressing mutual authentication scheme for smart IoT home network. *Multim. Tools Appl.* **84**, 25111–25143. 10.1007/s11042-024-19898-y (2025).

